# Post-transcriptional regulation of fruit ripening and disease resistance in tomato by the vacuolar protease SlVPE3

**DOI:** 10.1186/s13059-017-1178-2

**Published:** 2017-03-07

**Authors:** Weihao Wang, Jianghua Cai, Peiwen Wang, Shiping Tian, Guozheng Qin

**Affiliations:** 1 Key Laboratory of Plant Resources, Institute of Botany, Chinese Academy of Sciences, No. 20 Nanxincun, Xiangshan, Haidian District, Beijing, 100093 China; 2 University of Chinese Academy of Sciences, Yuquanlu, Beijing, 100049 China

**Keywords:** Fruit ripening, Disease resistance, Protease, Vacuolar processing enzyme, RNA interference, Quantitative proteome, Protease inhibitor, Tomato

## Abstract

**Background:**

Proteases represent one of the most abundant classes of enzymes in eukaryotes and are known to play key roles in many biological processes in plants. However, little is known about their functions in fruit ripening and disease resistance, which are unique to flowering plants and required for seed maturation and dispersal. Elucidating the genetic mechanisms of fruit ripening and disease resistance is an important goal given the biological and dietary significance of fruit.

**Results:**

Through expression profile analyses of genes encoding tomato (*Solanum lycopersicum*) cysteine proteases, we identify a number of genes whose expression increases during fruit ripening. RNA interference (RNAi)-mediated repression of *SlVPE3*, a vacuolar protease gene, results in alterations in fruit pigmentation, lycopene biosynthesis, and ethylene production, suggesting that *SlVPE3* is necessary for normal fruit ripening. Surprisingly, the *SlVPE3* RNAi fruit are more susceptible to the necrotrophic pathogen *Botrytis cinerea*. Quantitative proteomic analysis identified 314 proteins that differentially accumulate upon *SlVPE3* silencing, including proteins associated with fruit ripening and disease resistance. To identify the direct SlVPE3 targets and mechanisms contributing to fungal pathogen resistance, we perform a screening of SlVPE3-interacting proteins using co-immunoprecipitation coupled with mass spectrometry. We show that SlVPE3 is required for the cleavage of the serine protease inhibitor KTI4, which contributes to resistance against the fungal pathogen *B. cinerea*.

**Conclusions:**

Our findings contribute to elucidating gene regulatory networks and mechanisms that control fruit ripening and disease resistance responses.

**Electronic supplementary material:**

The online version of this article (doi:10.1186/s13059-017-1178-2) contains supplementary material, which is available to authorized users.

## Background

Fruit are highly specialized plant organs that play a central role in seed maturation and dispersal in angiosperms. They are also valuable components of human diets, providing essential nutrients and a wide range of “bioactive” compounds that are important for human health [1]. The ripening of fleshy fruit is a genetically programmed process that is associated with changes in color, texture, flavor, and susceptibility to pathogen infection [2, 3]. Substantial insights have been made into the mechanistic basis of ripening, including its regulation by transcription factors and the gaseous hormone ethylene, which plays a central role in the ripening of climacteric fruit [4, 5]. The ethylene biosynthetic and signal transduction pathway have been well characterized [6–8]. Recently, significant progress has been made in understanding the transcriptional control of fruit ripening using tomato (*Solanum lycopersicum*) as a model system. The transcription factors RIPENING-INHIBITOR (RIN) [9], NONRIPENING (NOR) [10], and COLORLESS NONRIPENING (CNR) [11] function as global regulators of ripening and act upstream of ethylene, while additional transcription factors that are required for normal ripening include TOMATO AGAMOUS-LIKE1 (TAGL1) [12, 13], HD-ZIP HOMEOBOX PROTEIN-1 (HB-1) [14], APETALA2a (AP2a) [15, 16], ETHYLENE RESPONSE FACTOR6 (ERF6) [17], ARABIDOPSIS PSEUDO RESPONSE REGULATOR2-LIKE (APRR2-Like) [18], and FRUITFULL (TDR4/FUL1 and MBP7/FUL2) [19]. In addition to transcriptional regulation, gene expression in fruit ripening can also be modulated by epigenetic or translational control [20–22]. However, unlike the relatively well studied and defined changes in the fruit transcriptome during ripening, little is known about its post-transcriptional regulation.

One mode of post-translational control involves proteases, enzymes that are widely distributed in all living organisms and that catalyze the hydrolysis of peptide bonds during or after translation [23]. Proteases specifically cleave protein substrates either from the N- or C- terminus (aminopeptidases and carboxypeptidases, respectively) or within the molecule (endopeptidases) [24]. Based on the active site residues or ions that carry out catalysis, proteases are generally grouped into five classes: aspartic, cysteine, metallo-, serine, and threonine proteases [25]. Although initially considered to be purely degradative enzymes involved in intracellular protein turnover, it is now clear that they participate in the regulation of many critical physiological and cellular processes [23, 25]. The information housed in the MEROPS peptidase database [26] suggests that a plant genome sequence encodes hundreds of proteases belonging to dozens of unrelated families. These enzymes have been associated with a wide variety of biological processes, including programmed cell death (PCD), meiosis, seed coat formation, cuticle deposition, stomata development, flowering, pollen or embryo development, and chloroplast biogenesis [27]. However, the molecular mechanisms of plant proteases in these processes, such as their substrates, remain elusive. Moreover, the functional roles of proteases in fruit ripening have not been well defined. Our previous study has shown that specific E2 ubiquitin-conjugating enzymes, the components of an ubiquitin-proteasome system, contribute to the ripening of tomato fruit [28]. From this we infer that proteolysis is involved in the regulation of fruit ripening, but how proteases participate in this process and the nature of the underlying mechanisms are not known.

In the study presented here, we identified a number of cysteine protease genes whose expression increases during tomato fruit ripening. We then assessed the physiological importance for fruit ripening of one of these genes, *SlVPE3*, which encodes a vacuolar processing enzyme (VPE). VPEs are canonical cysteine proteases, harboring active cysteines in the catalytic site, and are responsible for the maturation or activation of specific vacuolar proteins in plants [29]. Here, we show that suppression of *SlVPE3* by RNA interference (RNAi) in tomato delays ripening-related traits, including lycopene accumulation and ethylene synthesis. Interestingly, the tomato fruit with reduced *SlVPE3* expression exhibited increased susceptibility to the necrotrophic pathogen *Botrytis cinerea*, despite showing a reduced ripening phenotype. This was unexpected since unripe green fruit are typically less susceptible than ripe fruit to pathogen infections [3, 30]. To investigate how SlVPE3 affects these processes, we performed a comparative proteomic analysis using isobaric tags for relative and absolute quantification (iTRAQ). This suggested a large number of proteins as potential SlVPE3 targets, including some involved in fruit ripening and disease responses. We also carried out a screen for SlVPE3-interacting proteins to identify the direct targets of SlVPE3. We provide evidence that a Kunitz trypsin inhibitor 4, a serine protease inhibitor, functions downstream of SlVPE3 to regulate disease resistance in tomato fruit.

## Results

### Expression of the protease *SlVPE3* increases steadily during fruit ripening

**Fig. 1:**
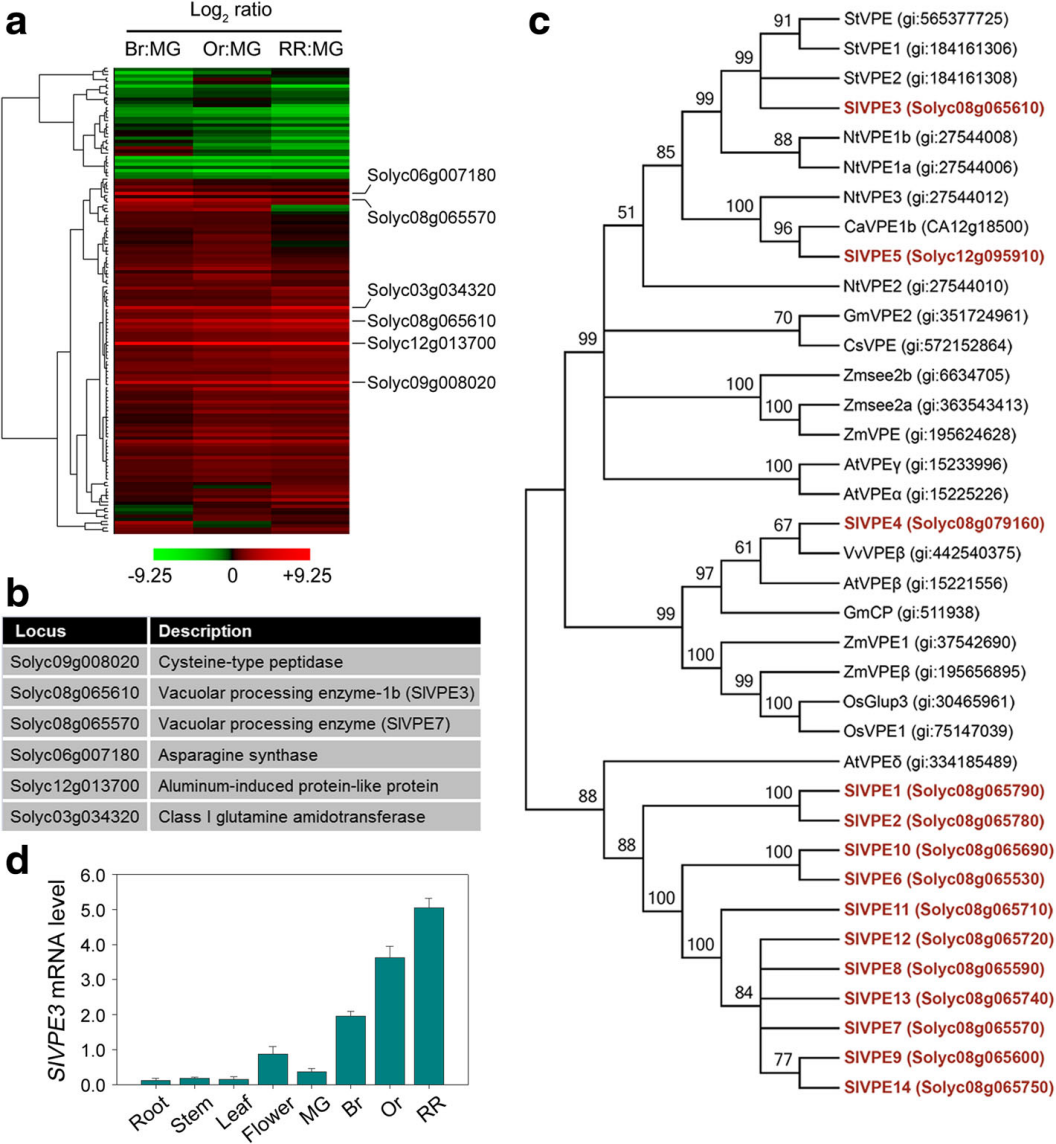
Expression analyses of tomato cysteine proteases reveal the involvement of *SlVPE3* in fruit ripening. **a** Expression profiles of tomato cysteine protease genes during fruit ripening, as determined by quantitative RT-PCR. The *ACTIN* gene was used as the internal control. The stages of fruit development analyzed were mature green (*MG*), breaker (*Br*), orange (*Or*), and red ripe (*RR*). Expression ratios were calculated using the earliest stage (*MG*) as the denominator and plotted in a heat map on a log_2_ scale. Each row in the color heat map represents a single cysteine protease gene. The *green* and *red colors* indicate down- and up-regulation, respectively, at an indicated ripening stage relative to the MG stage. *Black* indicates no significant expression change. Data from biologically repeated samples are averaged and the detailed information is listed in Additional file 2: Table S2. The genes whose mRNA levels increased more than tenfold are shown. **b** Gene identifiers (Solyc numbers) and functional annotations of the cysteine protease genes whose mRNA levels increased more than tenfold during tomato fruit ripening as revealed by quantitative RT-PCR. **c** Phylogenetic analysis of plant vacuolar proteases. The phylogenetic tree was produced using MEGA version 5.2. Bootstrap values from 1000 replications for each branch are shown. Tomato proteins are indicated in *red*. Species names are abbreviated as follows: St, *Solanum tuberosum*; Sl, *S. lycopersicum*; Nt, *Nicotiana tabacum*; Ca, *Capsicum annuum*; Gm, *Glycine max*; Cs, *Citrus sinensis*; Zm, *Zea mays*; At, *Arabidopsis thaliana*; Vv, *Vitis vinifera*; Os, *Oryza sativa*. The accession numbers are indicated in *parentheses*. **d** Gene expression of *SlVPE3* in vegetative and reproductive tomato organs as determined by quantitative RT-PCR. The *ACTIN* gene was used as an internal control. Values are means ± standard deviation of three independent experiments

The tomato genome encodes more than 900 predicted proteases of diverse catalytic classes, based on the MEROPS protease database [26], but in this study we focused on cysteine proteases, a class that has been shown to take part in a variety of biological processes [27]. A total of 167 non-redundant cysteine proteases, belonging to 19 families, were identified from the tomato genome using the MEROPS database (Additional file 1: Table S1). Expression analysis of the corresponding genes using quantitative reverse transcriptase PCR (RT-PCR) (Additional file 2: Table S2) and hierarchical clustering analysis [31] revealed several genes whose transcript levels increased during fruit ripening (Fig. 1a). Those whose expression increased more than tenfold are shown in Fig. 1a, b. Of these, two encoded VPEs, a class of proteins that were originally identified as cysteine proteases responsible for the maturation of seed storage proteins [32]. They were later reported to be the plant functional orthologs of animal caspases, which are essential for the initiation and execution of PCD [29, 33, 34]. In addition, the transcript levels of a *VPE* gene from *Citrus sinensis* have been observed to increase during fruit ripening [35], which when taken together with our results suggests that VPE proteins might contribute to ripening in a range of species.

According to the MEROPS protease database, the tomato genome has 14 *VPE* genes, five of which have previously been identified and named *SlVPE1* to *SlVPE5* [36]. We named the other nine *VPE* genes *SlVPE6* to *SlVPE14* on the basis of their chromosomal location (Additional file 3: Table S3). All these VPE proteins are predicted to contain two conserved cysteine residues in the active sites (Additional file 4: Figure S1). Phylogenetic analysis revealed that tomato VPE proteins can be divided into several subgroups, with >50% bootstrap support (Fig. 1c), and high sequence similarity among the proteins was observed (Additional file 5: Table S4), suggesting gene duplications. We selected *SlVPE3* for functional analysis because its expression was not only higher in fruit than in other organs, such as root, stem, and leaf, but also increased gradually during fruit ripening (Fig. 1d). *SlVPE3* has been shown to be involved in controlling sugar accumulation [36], but its function in fruit ripening and the underlying molecular mechanisms are unclear.

### *SlVPE3* is required for tomato fruit ripening

**Fig. 2:**
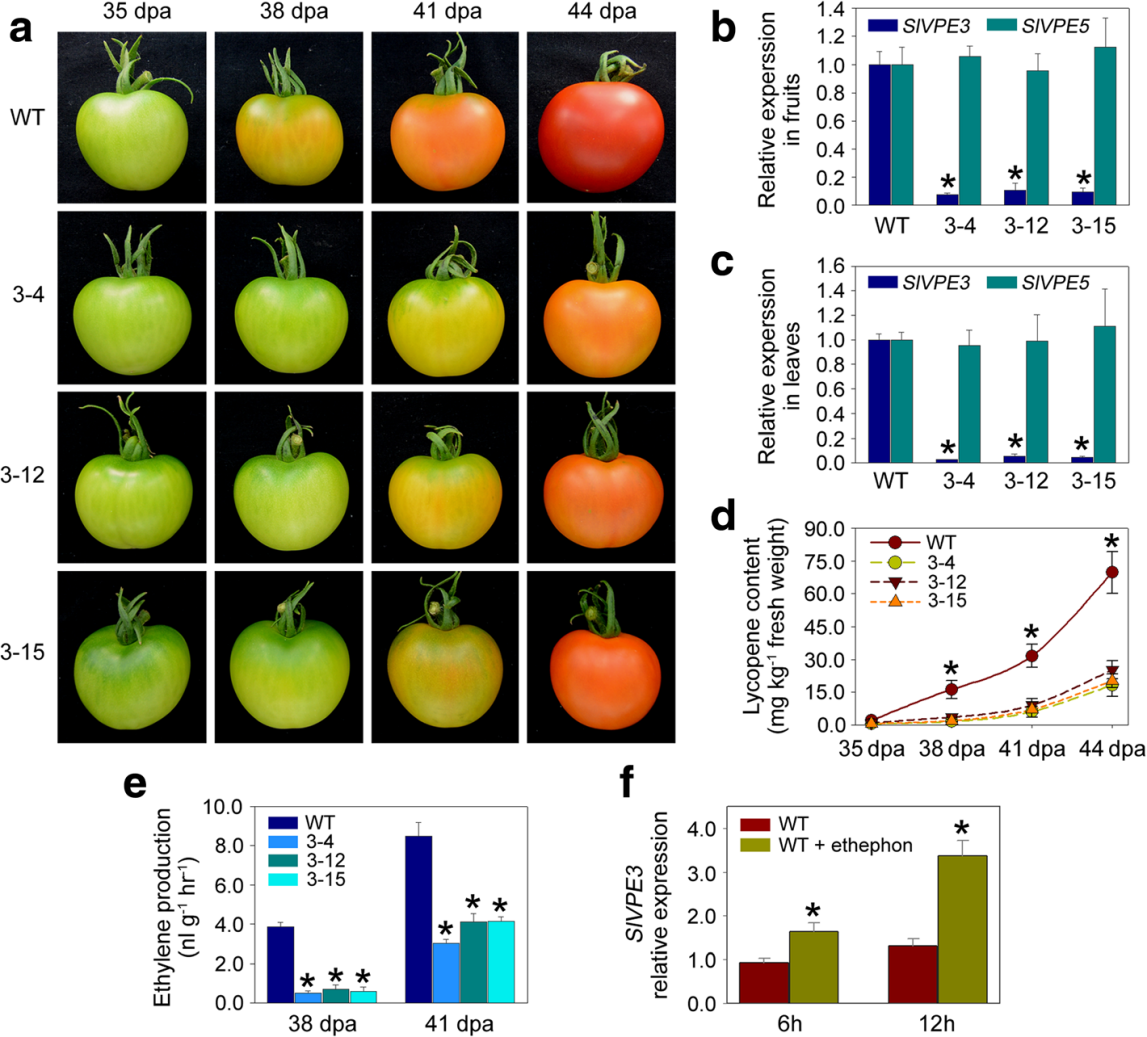
*SlVPE3* is necessary for normal tomato fruit ripening. **a** Ripening phenotype of *SlVPE3* RNAi lines. Fruit at 35, 38, 41, and 44 days post-anthesis (*dpa*) from wild-type (*WT*) and *SlVPE3* RNAi lines (3-4, 3-12, and 3-15) are shown. **b** Expression of *SlVPE3* and *SlVPE5* in fruit of WT and *SlVPE3* RNAi lines as determined by quantitative RT-PCR. **c** Expression of *SlVPE3* and *SlVPE5* in leaves of WT and *SlVPE3* RNAi lines. In **b** and **c**, the gene transcript levels were normalized against the *ACTIN* gene, followed by normalization against WT expression. Values are shown as the means ± standard deviation (SD). *Asterisks* indicate *P* value <0.05 (*t*-test) when comparing values for each measurement between the *SlVPE3* RNAi lines and WT plants. **d** Lycopene accumulation in WT and *SlVPE3* RNAi fruit during ripening. **e** Ethylene generation in WT and *SlVPE3* RNAi fruit at 38 and 41 dpa. In **d** and **e**, values are shown as the means ± SD. *Asterisks* indicate significant differences (*P* < 0.05; *t*-test) between WT and *SlVPE3* RNAi fruit at an indicated ripening stage. **f** Expression of *SlVPE3* in WT fruit (35 dpa) 6 or 12 h after untreated or treated with ethephon, as determined by quantitative RT-PCR. Gene transcript levels were normalized against expression of the *ACTIN* gene, followed by normalization against expression in the WT without ethephon treatment. Values are shown as the means ± SD. *Asterisks* indicate significant differences (*P* < 0.05; *t*-test) at an indicated time point after treatment

To gain insight into the function of *SlVPE3*, we generated a *SlVPE3* RNAi construct under the control of a 35S cauliflower mosaic virus promoter and transformed it into the wild-type tomato cultivar Ailsa Craig. Three independent transgenic lines (3-4, 3-12, and 3-15) with confirmed transgene integration showed distinct and similar ripening-related phenotypes (Fig. 2a). The differences in fruit ripening between the *SlVPE3* RNAi lines and wild-type became apparent at 38 days post-anthesis (dpa). A visible color change could be observed at this stage in the wild-type fruit, whereas *SlVPE3* RNAi tomatoes were still green. At 41 dpa, the wild-type fruit had a homogenous orange color, while fruit from the *SlVPE3* RNAi lines were only just starting to change color. To verify the specific repression of *SlVPE3* in the RNAi lines, total RNA was extracted from fruit and leaves of wild-type and transgenic lines and submitted to quantitative RT-PCR analysis. The transcript levels of *SlVPE3* were shown to be strongly reduced in both organs of transgenic lines compared with the wild-type (Fig. 2b, c). Sixteen potential off-targets for the RNAi construct were identified (Additional file 4: Figure S2) using the computational tool pssRNAit [37]. However, quantitative RT-PCR analysis indicated that none of these showed reduced expression in leaves of the *SlVPE3* RNAi lines (Additional file 4: Figure S2). This result indicated that the RNAi construct was specific for the target gene. We also measured the expression of *SlVPE5* because it is the most closely related tomato gene to *SlVPE3* (Fig. 1c). The levels of *SlVPE5* mRNA in all three RNAi lines (3-4, 3-12, and 3-15) were not significantly different from wild type (Fig. 2b, c), demonstrating the specificity of the *SlVPE3* RNAi construct for the target gene. The three lines (3-4, 3-12, and 3-15) were selected for further analysis.

The color change of ripening tomatoes from green to red is largely due to the degradation of chlorophyll and the accumulation of lycopene, which accounts for 70–90% of the total carotenoids in most tomato varieties [38, 39]. To establish the underlying causes of the color differences observed between wild-type and *SlVPE3* RNAi ripe fruit, we measured the levels of lycopene. As shown in Fig. 2d, levels of lycopene in fruit from the *SlVPE3* RNAi lines were <10% of wild-type levels at 38 dpa, suggesting that *SlVPE3* expression affects lycopene accumulation during fruit ripening.

As with all climacteric fruit, those of tomato require an increase in ethylene biosynthesis to ripen [4]. We investigated whether the delay in fruit ripening in the *SlVPE3* RNAi lines correlated with ethylene production. It was observed that fruit of the repressed lines (3-4, 3-12, and 3-15) produced less ethylene than wild-type fruit at the same ripening stages (Fig. 2e). Ethylene regulates the expression of many genes associated with fruit ripening [40] and we observed that the expression of *SlVPE3* increased during tomato fruit ripening (Fig. 1d). To determine whether the increased expression of *SlVPE3* was ethylene-dependent, wild-type fruit at 35 dpa were treated with the ethylene precursor ethephon, which resulted in a substantial increase in expression (Fig. 2f). Taken together, these results suggest that ethylene induces the expression of *SlVPE3*, which in turn regulates ethylene synthesis in tomato fruit by a positive feedback loop.

### Disease resistance is impaired in *SlVPE3* silenced fruit

**Fig. 3:**
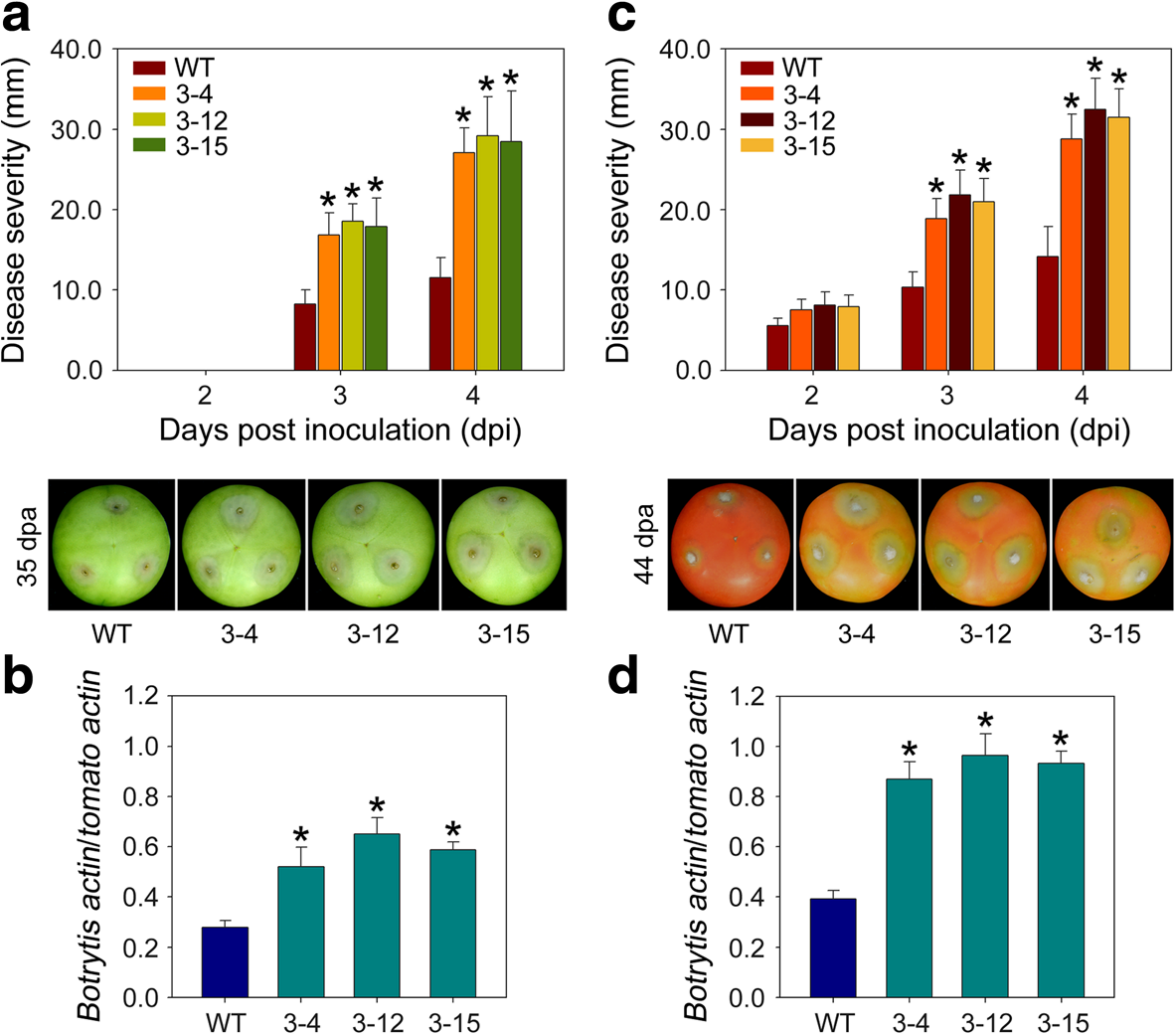
Suppressing *SlVPE3* enhances susceptibility of tomato fruit to *B. cinerea*. **a** Disease severity for fruit inoculated with *B. cinerea* at 35 days post-anthesis (*dpa*). Representative fruit at 3 days post-inoculation (*dpi*) for wild-type (*WT*) and *SlVPE3* RNAi lines (3-4, 3-12, and 3-15) are shown. **b** Fungal growth during infection of tomato fruit at 35 dpa. Growth of *B. cinerea* at 3 dpi in infected WT and *SlVPE3* RNAi fruit was measured based on expression levels of *B. cinerea ACTIN 2* relative to tomato *ACTIN* gene. **c** Disease severity in fruit inoculated with *B. cinerea* at 44 dpa. Representative fruit at 3 dpi for WT and *SlVPE3* RNAi lines (3-4, 3-12, and 3-15) are shown. **d** Fungal growth during infection of tomato fruit at 44 dpa. Growth of *B. cinerea* at 3 dpi in infected WT and *SlVPE3* RNAi fruit was measured based on quantitative PCR amplification of *B. cinerea ACTIN 2* relative to tomato *ACTIN* gene. Values are shown as the means ± standard deviation. *Asterisks* indicate significant differences (*P* < 0.05; *t*-test) between WT and *SlVPE3* RNAi fruit at a given time point after inoculation

Fruit ripening involves the regulation of numerous biochemical pathways associated with pigmentation, cell wall metabolism, and the production of aromatic and nutritionally important compounds [13]. Additionally, ripening is characterized by a major increase in susceptibility to necrotrophic pathogens [3, 30]. Since repression of *SlVPE3* resulted in a delay in fruit ripening, we investigated whether fruit from the *SlVPE3* RNAi lines were also less susceptible to pathogen infection. Fruit at 35 and 44 dpa were inoculated with *B. cinerea*, a commercially important tomato postharvest pathogen that causes gray mold disease [30]. As shown in Fig. 3a, soft rot symptoms occurred at the third day post-inoculation in the fruit of both wild-type and the *SlVPE3* RNAi lines (3-4, 3-12, and 3-15) at 35 dpa. An approximately twofold increase in lesion development was observed in *SlVPE3* RNAi fruit compared with the wild type. Fungal growth, assessed based on the quantitative PCR amplification of *B. cinearea ACTIN 2* (BC1G_08198), was significantly greater in the *SlVPE3* RNAi fruit (Fig. 3b). In contrast, tissue rotting was evident on the second day post-inoculation in wild-type and *SlVPE3* RNAi fruit at 44 dpa. Similarly, a significant increase in disease severity and fungal growth was observed in the *SlVPE3* RNAi fruit (Fig. 3c, d). These data indicated that silencing *SlVPE3* expression makes the fruit more susceptible to pathogen infection, even though fruit ripening was delayed. Since this finding was counter-intuitive and contradictory to previous observations that unripe fruit are usually less susceptible than ripe fruit to infection [3, 30], we hypothesized that SlVPE3 may target downstream proteins associated with pathogen resistance in tomato fruit.

### Quantitative proteome analysis identifies proteins that differentially accumulate upon *SlVPE3* silencing

**Fig. 4:**
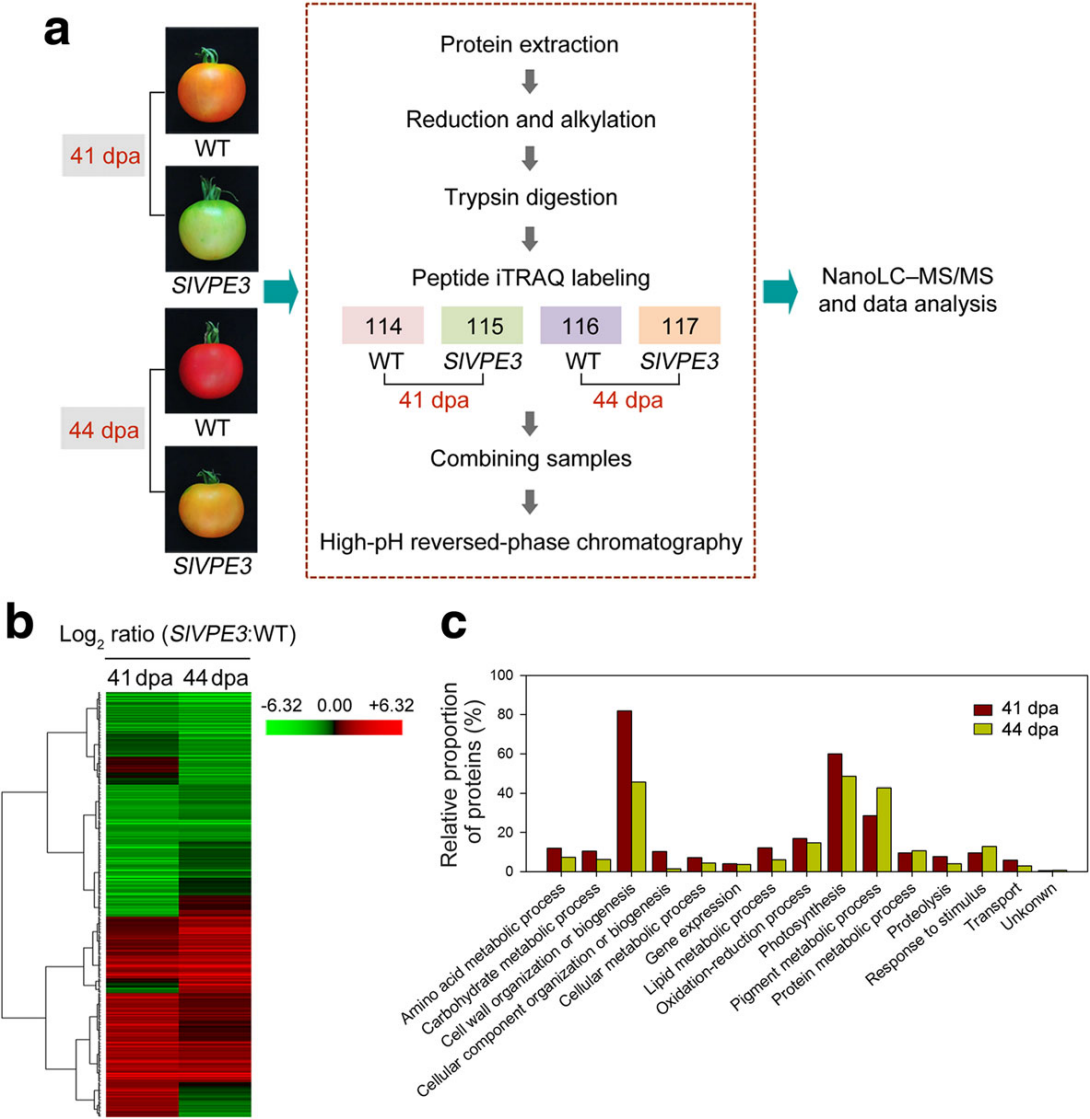
Quantitative proteome analysis reveals the potential targets of SlVPE3. **a** Workflow of the quantitative proteome analysis. Proteins were isolated from wild-type (*WT*) and *SlVPE3* RNAi fruit at 41 or 44 days post-anthesis (*dpa*), and subjected to isobaric tags for relative and absolute quantification (*iTRAQ*) labeling coupled with nanoLC–MS/MS. **b** A total of 314 proteins showing differential expression in the *SlVPE3* RNAi fruit compared to the WT were identified. The expression patterns of the proteins were hierarchically clustered based on the expression ratio as a log_2_ scale. Each row in the color heat map represents a single protein. The *green* and *red colors* indicate down- and up-regulation, respectively, in the *SlVPE3* RNAi fruit relative to the WT. *Black* represents no significant expression change. Values from biological replicates were averaged and the detailed information associated with the identified proteins is listed in Additional file 6: Table S5. **c** Functional categories of the proteins that changed abundance in the *SlVPE3* RNAi fruit at 41 or 44 dpa compared with WT

Protease target identification is essential to understand the molecular mechanism by which it regulates biological processes. An iTRAQ-based quantitative proteomic approach was employed to identify the proteins that differentially accumulate upon *SlVPE3* silencing. The general design of the experiment and experimental workflow is shown in Fig. 4a. Proteins were isolated from *SlVPE3* RNAi fruit (T1 generation) and wild-type fruit at 41 and 44 dpa and labeled with 4-plex iTRAQ reagents. The iTRAQ experiment was repeated with an independent biological replicate. For each replicate, pericarp tissue of fruit from five plants was pooled to account for variation among individuals. Using the *S. lycopersicum* protein database ITAG2.4_proteins_full_desc.fasta, a total of 3802 and 3840 proteins were identified in biological replicates 1 and 2, respectively, with a global false discovery rate (FDR) of <1% in both. A twofold cut-off was then used as a determinant for whether the changes in protein abundance were significant. The levels of 314 proteins were significantly different in the *SlVPE3* RNAi fruit compared with those of the wild type at one ripening stage or both. Additional file 6: Table S5 lists these proteins, along with the associated identification information and the ratio of iTRAQ reporter ion intensities. To identify the proteins with similar expression profiles, hierarchical clustering [31] was applied and the 314 proteins were divided into two main clusters, representing those that had higher or lower abundance in the *SlVPE3* RNAi fruit than in wild type (Fig. 4b). The Blast2go [41] web-based bioinformatic tool, which categorizes proteins according to their Gene Ontology annotations, classified the proteins into 15 functional categories (Fig. 4c). The functional classes “cell wall organization or biogenesis”, “photosynthesis”, and “pigment metabolic process” contained the largest number of proteins at both ripening stages (41 and 44 dpa).

### SlVPE3 modulates the accumulation of proteins associated with fruit ripening and disease resistance

**Fig. 5:**
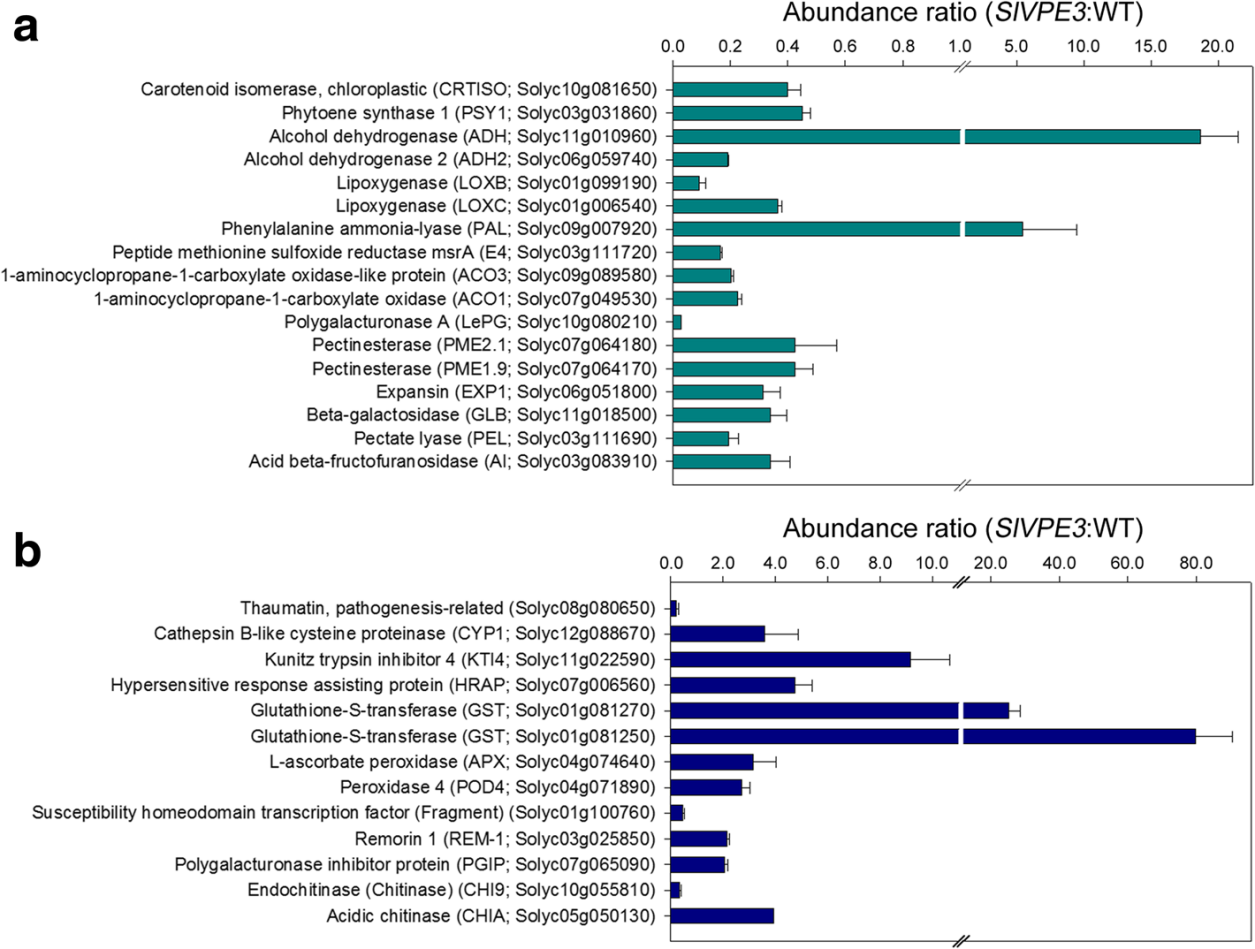
*SlVPE3* RNAi fruit showed altered levels of proteins associated with fruit ripening and disease resistance. Proteins isolated from wild-type (*WT*) and *SlVPE3* RNAi fruit were subjected to isobaric tags for relative and absolute quantification (*iTRAQ*) labeling coupled with nanoLC–MS/MS. The abundance ratio of proteins in the *SlVPE3* RNAi fruit versus WT at 41 days post-anthesis (*dpa*) is shown. Values represent means of two biological replicates, and *error bars* represent standard deviation. **a** Identification of proteins associated with fruit ripening in the iTRAQ analysis. **b** Identification of proteins associated with disease resistance in the iTRAQ analysis. The gene identifiers (Solyc numbers) and the functional annotations are indicated. Detailed protein information is listed in Additional file 6: Table S5

The proteins associated with fruit ripening that showed significant differences in abundance in the *SlVPE3* RNAi fruit compared with wild type included some involved in cell wall degradation, such as polygalacturonase A (LePG, Solyc10g080210); ethylene synthesis, such as 1-aminocyclopropane-1-carboxylate oxidase (ACO1, Solyc07g049530); aroma formation, such as lipoxygenase (LoxC, Solyc01g006540); and carotenoid biosynthesis, such as phytoene synthase 1 (PSY1, Solyc03g031860) (Fig. 5a). The levels of most of these proteins were lower in the *SlVPE3* RNAi fruit, consistent with the delayed ripening phenotype. We also identified a set of proteins associated with disease responses, such as endochitinase (CHI9, Solyc10g055810), polygalacturonase inhibitor protein (PGIP, Solyc07g065090), and thaumatin (Solyc08g080650) (Fig. 5b), although among proteins were examples with either decreased or increased abundance in the *SlVPE3* RNAi fruit. Strikingly, the list of differentially abundant proteins included acid beta-fructofuranosidase (AI, Solyc03g083910), the tomato ortholog of AtFruct4, which is a downstream target of *Arabidopsis thaliana* VPEγ [29]. This highlighted the value of quantitative proteome analysis for identifying putative protease targets. It should be noted that, besides SlVPE3 targets, we also identified other proteins that differentially accumulated due to the delayed ripening.

### SlVPE3 affects gene transcript levels indirectly

**Fig. 6:**
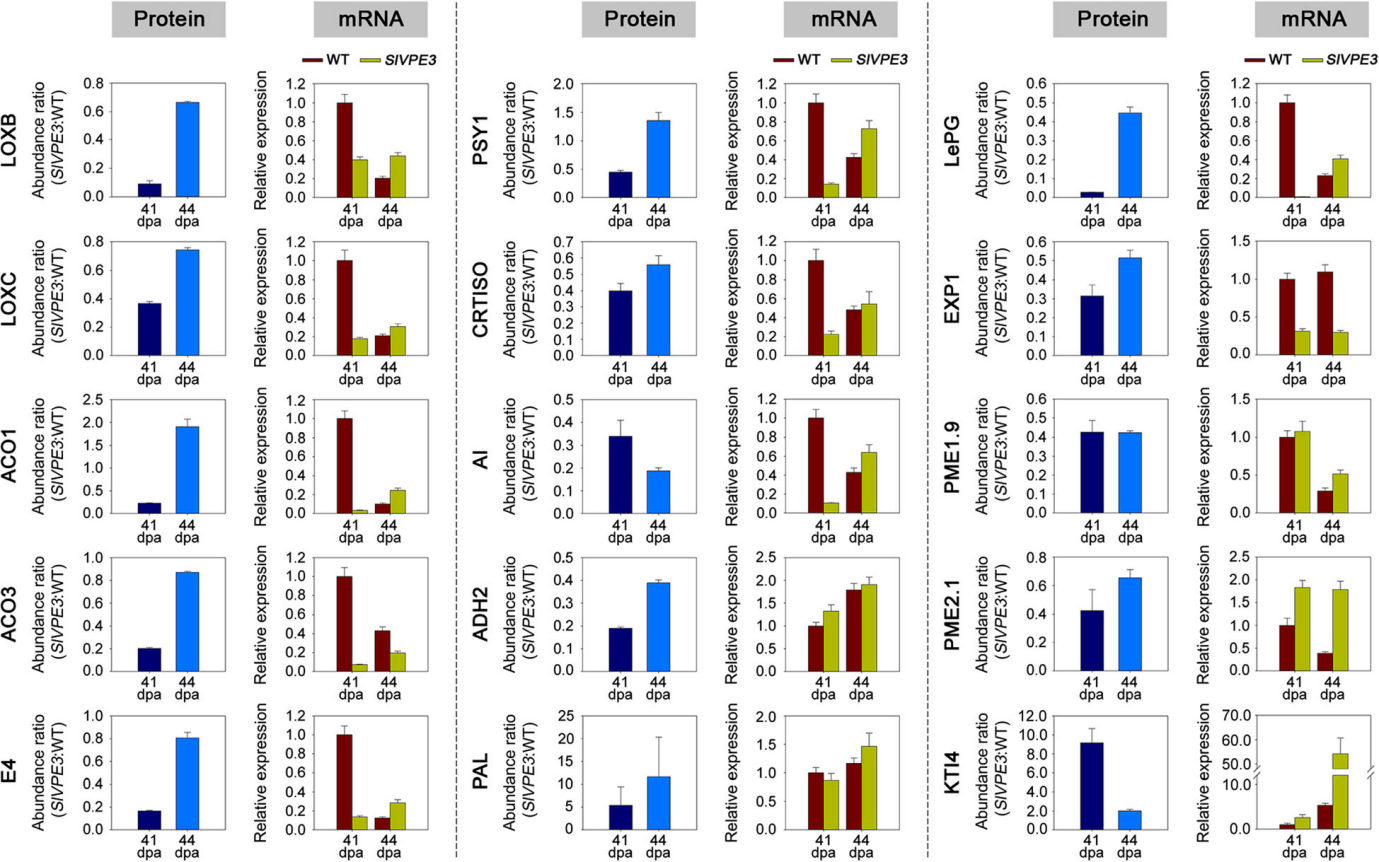
Comparison of expression profiles at the protein and mRNA levels. The protein abundance in wild-type (*WT*) and *SlVPE3* RNAi fruit at 41 and 44 days post-anthesis (dpa) was assessed by isobaric tags for relative and absolute quantification (iTRAQ)-based quantitative proteome analysis. Transcript abundance was evaluated by quantitative RT-PCR. The gene transcript levels are normalized against the *ACTIN* gene, followed by normalization against the expression in WT. Values are shown as the means ± standard deviation

**Fig. 7:**
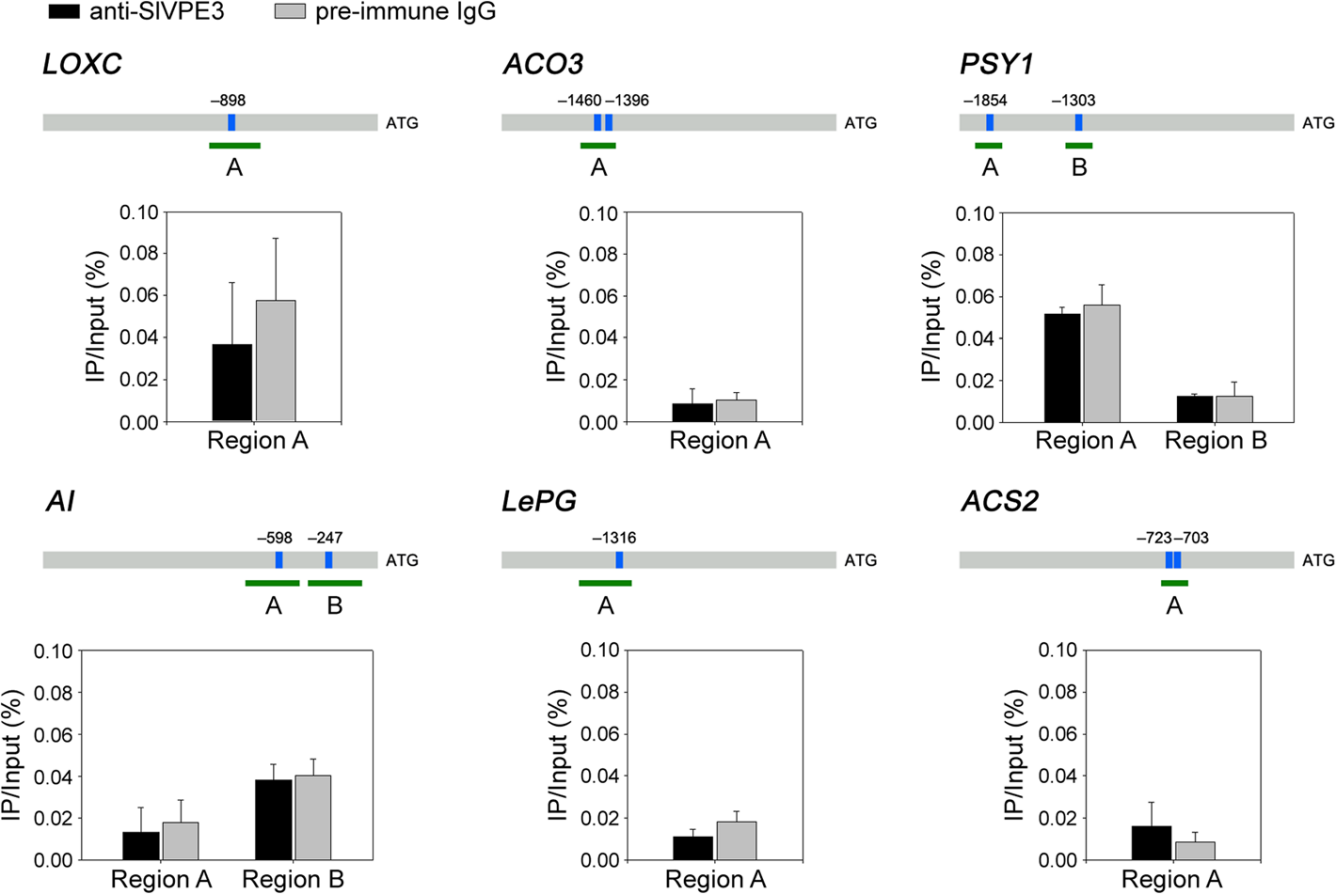
SlVPE3 does not bind to the promoters of genes with abnormal expression in *SlVPE3* silenced fruit. For the ChIP assay, genomic DNA and proteins from pericarp of tomato fruit at 41 dpa were cross-linked, and the chromatin complexes were co-immunoprecipitated with anti-SlVPE3 antibodies. The promoter regions of the analyzed genes are indicated. *Blue boxes* represent the binding motifs and *numbers* indicate the position of these motifs relative to the translational start site. The *green fragments with upper-case letters* represent the regions used for ChIP-quantitative PCR. Values represent the percentage of DNA fragments that were co-immunoprecipitated with anti-SlVPE3 antibodies or preimmune serum (rabbit IgG) relative to the input DNA. *Error bars* represent the standard deviation of three independent experiments

A previous study demonstrated that SlVPE5 (formerly named LeCp), a paralog of SlVPE3, can act as a transcription factor [42]. LeCp was reported to specifically bind to the promoter of *ACC SYNTHASE 2* (*ACS2*), a key gene in ethylene biosynthesis, and this binding correlated induction of *ACS2* in tomato leaves by the fungal elicitor Ethylene-inducing xylanase (EIX) [42]. To determine whether SlVPE3 acts as a transcription factor, we first investigated whether the protein expression patterns observed in the quantitative proteomic analysis correlated with cognate transcript levels. Of the 15 genes selected for quantitative RT-PCR analysis, the expression of 11, including *LoxC*, *ACO1*, *E4*, *PSY1*, *CRTISO*, and *LePG*, was consistent with the variations in protein abundance (Fig. 6). Next, we performed a chromatin immunoprecipitation (ChIP) assay to investigate whether SlVPE3 could bind directly to the promoters of these genes. A search for the putative DNA binding site in the 2000-bp upstream regions starting from the translational start sites revealed that only five of the genes (*LoxC*, *ACO3*, *PSY1*, *AI*, and *LePG*) contained the putative TAAAATAT binding motif [42] (Additional file 7: Table S6). For the ChIP assay, the cross-linked DNA–protein complexes were immunoprecipitated using an affinity-purified polyclonal antibody raised against SlVPE3. Specific primers (Additional file 8: Table S7) were designed for the five genes (*LoxC*, *ACO3*, *PSY1*, *AI*, and *LePG*) to amplify promoter sequences surrounding the putative binding sites from the immunoprecipitated DNA. We were not able to detect any specific enrichment for the promoters of these genes (Fig. 7). We also did not observe an enrichment of the *ACS2* promoter (Fig. 7), although it has been shown to be bound by LeCp [42]. These data suggest that SlVPE3 affects gene transcript levels indirectly and so SlVPE3 may function at the post-transcriptional level, rather than directly regulating gene expression as a transcription factor.

### Screening for SlVPE3-interacting proteins

**Fig. 8:**
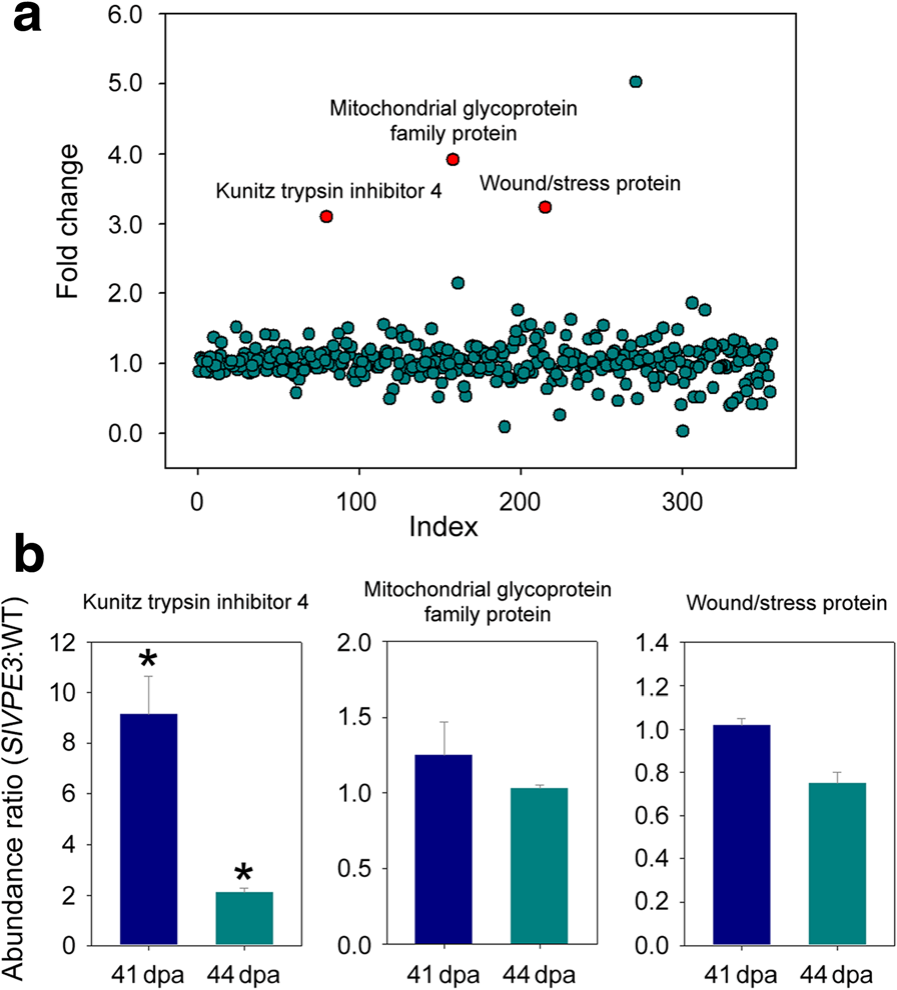
Identification of endogenous SlVPE3-interacting proteins. **a** Proteins isolated from tomato fruit at 41 days post-anthesis (*dpa*) were immunoprecipitated with anti-SlVPE3 antibodies or pre-immune serum IgG (negative control). The enriched proteins were eluted and submitted to Sequential Window Acquisition of all Theoretical Mass Spectra (SWATH-MS) analysis. Each *circle* represents a single protein. Most identified proteins showed no difference in abundance, indicating nonspecifically bound proteins. Three proteins (*red circle*) were identified as the SlVPE3-interacting proteins with significant increases in abundance (*P* < 0.01; *t*-test) by using anti-SlVPE3 antibodies compared with pre-immune serum IgG. Additional information related to protein identification is listed in Additional file 9: Table S8. **b** The changes in protein abundance in the *SlVPE3* RNAi fruit revealed by isobaric tags for relative and absolute quantification (iTRAQ) analysis. The abundance ratios of selected proteins in the *SlVPE3* RNAi fruit versus wild-type (*WT*) at 41 and 44 dpa are shown. Values represent means of two biological replicates, and error bars represent standard deviation. *Asterisks* indicate significant differences at a twofold cut-off between WT and *SlVPE3* RNAi fruit

To identify the direct targets of SlVPE3, we screened for SlVPE3-interacting proteins by analyzing proteins that were immunoprecipitated with affinity-purified anti-SlVPE3 polyclonal antibody or pre-immune serum IgG (non-specific antibody; negative control) from 41-dpa wild-type fruit. The immunoprecipitated proteins were submitted to Sequential Window Acquisition of all Theoretical Mass Spectra (SWATH-MS) quantitative proteomic analysis [43], which measures quantitative changes in protein interactions [44]. This resulted in the identification of 355 proteins, of which 57 were also identified by the iTRAQ proteome analysis as showing significantly different abundance in *SlVPE3* RNAi fruit compared with wild type (Additional file 9: Table S8). Most of the identified proteins had fold changes ~1 (Fig. 8a; Additional file 9: Table S8), indicating proteins that were immunoprecipitated regardless of whether anti-SlVPE3 or IgG was used, and so these proteins were considered to be nonspecific contaminants. Proteins with significantly increased abundance (*P* < 0.01) by using anti-SlVPE3 compared with pre-immune serum IgG included a mitochondrial glycoprotein family protein (Solyc03g079930), a wound/stress protein (Solyc03g093360), and a Kunitz trypsin inhibitor 4 (Solyc11g022590) (Fig. 8a), and these were designated as SlVPE3-interacting proteins. The Kunitz trypsin inhibitor 4 (KTI4) exhibited a significant difference in protein expression levels in *SlVPE3* RNAi fruit compared with wild type in the iTRAQ analysis (Fig. 8b), further suggesting that KTI4 might serve as a substrate of SlVPE3. Notably, SlVPE3 itself was not identified in the SWATH-MS analysis, which we hypothesized was due to the experimental conditions used for the elution of the immunoprecipitated proteins. Indeed, when we changed the conditions, SlVPE3 was identified as an immunoprecipitated protein (Additional file 4: Figure S3).

We also performed a yeast two-hybrid (Y2H) screen to identify proteins that interact with SlVPE3 using the Matchmaker Library Construction and Screening Kits (Clontech). SlVPE3 was used as bait against a tomato cDNA library constructed from wild-type fruit at the breaker stage (38 dpa). A total of 179 positive colonies were analyzed, leading to the identification of 49 proteins (Additional file 10: Table S9) involved in various cellular processes. Notably, several proteins associated with proteolysis were identified, including KTI4, which was then targeted for further analysis.

### SlVPE3 protein interacts directly with KTI4 in vacuoles

**Fig. 9:**
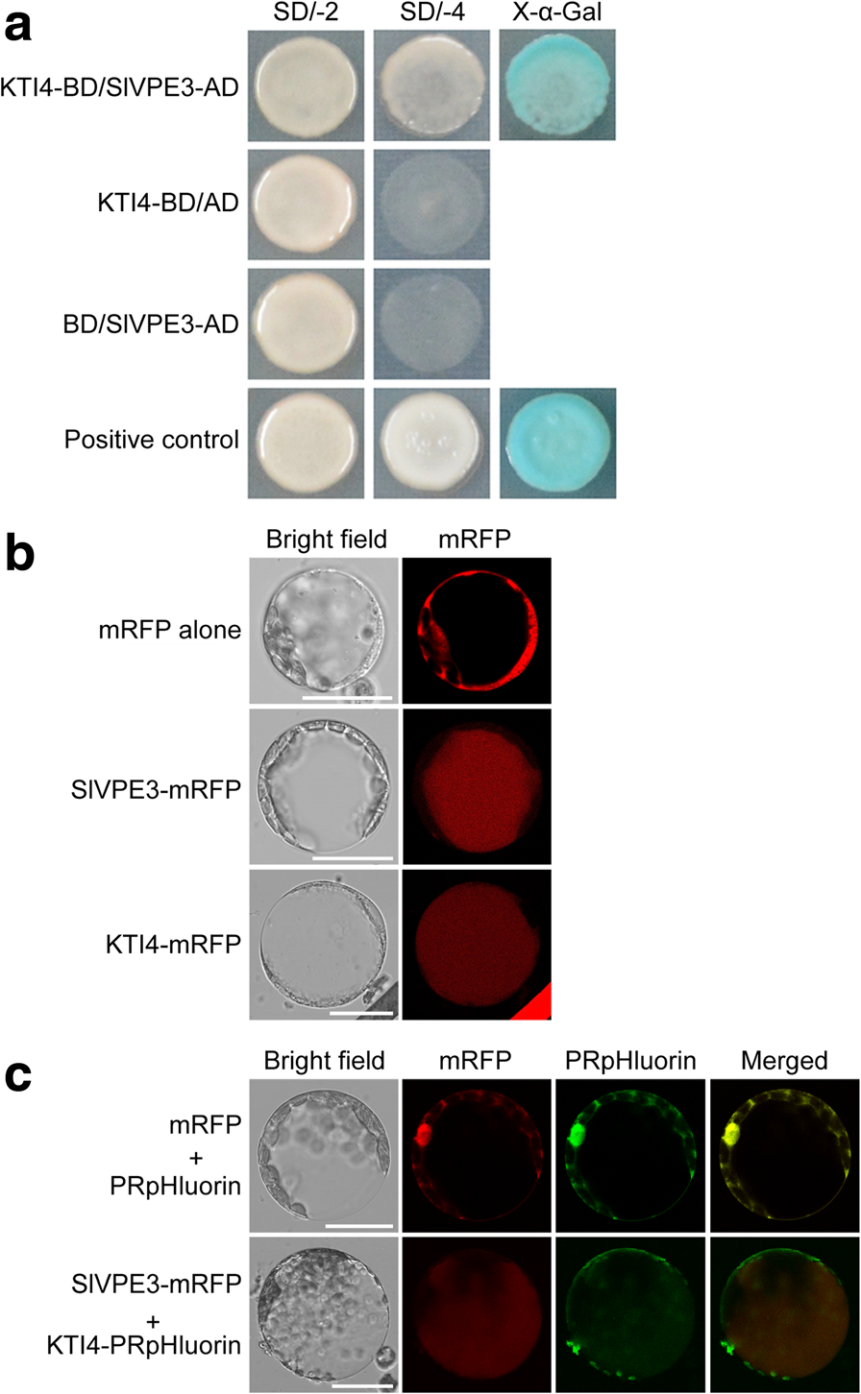
SlVPE3 interacts with KTI4 in vacuoles. **a** Interactions between KTI4 and SlVPE3 in a Y2H analysis. The ORF of *KTI4* and the cDNA fragment encoding the mature protein of *SlVPE3* were cloned into the pGBKT7 (BD) and pGADT7 (AD) vectors, respectively, resulting in the KTI4-BD and SlVPE3-AD plasmids, which were co-transformed into yeast. As negative controls, KTI4-BD and AD or SlVPE3-AD and BD were co-transformed into yeast. The transformants were streaked on SD/-Leu/-Trp medium (SD/-2). Protein–protein interactions were assessed by examining growth on SD/-Leu/-Trp/-His/-Ade medium (SD/-4) and further confirmed by monitoring β-galactosidase activity (*blue coloration*). **b** Subcellular localization of SlVPE3 and KTI4 visualized by monomeric *red* fluorescent protein (*mRFP*) analysis. The constructs used for transformation are indicated (*left*): *mRFP alone*, control showing the signals throughout the cell except in the vacuolar lumen; *SlVPE3-mRFP*, signals from the SlVPE3-mRFP fusion protein; *KTI4-mRFP*, signals from the KTI4-mRFP fusion protein. Protoplasts of tobacco (*Nicotiana benthamiana*) leaves transiently expressing the mRFP-alone control, SlVPE3-mRFP, or KTI4-mRFP were isolated and observed under a Leica confocal microscope (Leica DMI600CS). **c** Subcellular colocalization of SlVPE3 and KTI4 determined using *N. benthamiana* leaf protoplasts co-expressing SlVPE3-mRFP and KTI4-PRpHluorin. The constructs used for transformation are indicated (*left*): *mRFP + PRpHluorin*, control showing the signals throughout the cell, except in the vacuolar lumen; *SlVPE3-mRFP + KTI4-PRpHluorin*, signals from SlVPE3-mRFP and KTI4-PRpHluorin fusion proteins. Colocalization is shown by merging mRFP and PRpHluorin images (*Merged*). Scale bars, 25 μm

KTI4 belongs to the KTI family, members of which include serine protease inhibitors responsible for defense responses in leaves [45, 46]. However, little is known about the regulation of *KTI* genes and their functions in fruit disease resistance. To confirm the putative interaction between KTI4 and SlVPE3, we carried out a Y2H analysis. The open reading frame (ORF) of *KTI4* and the cDNA fragment encoding the mature protein of *SlVPE3* were cloned into the pGBKT7 (binding domain, BD) and pGADT7 (activation domain, AD) vectors, respectively. The resulting plasmids KTI4-BD and SlVPE3-AD were co-transformed into yeast, while yeast lines co-transformed with KTI4-BD and empty AD (KTI4-BD/AD) or SlVPE3-AD and empty BD (BD/SlVPE3-AD) were used as negative controls. KTI4-BD and SlVPE3-AD co-transformed yeast lines displayed normal growth and blue coloration when grown on the selective SD/-Leu/-Trp/-His/-Ade (SD/-4) medium containing X-α-Gal, whereas the control lines did not grow (Fig. 9a), further indicating that KTI4 interacts with SlVPE3.

To examine the intracellular localization of SlVPE3 and KTI4, their ORFs were separately introduced into a vector to generate a translational fusion with monomeric red fluorescent protein (mRFP) at the C-terminus. The constructs were separately introduced into *Agrobacterium tumefaciens*, which in turn was used to transform tobacco (*Nicotiana benthamiana*) leaves, from which mesophyll protoplasts were isolated. Protoplasts expressing mRFP alone served as a control. Confocal laser scanning microscopy showed that mRFP-tagged SlVPE3 (SlVPE3-mRFP) and KTI4 (KTI4-mRFP) both produced a strong signal in the vacuole, while the mRFP-only control displayed a fluorescent signal throughout the cell, but not from the vacuolar lumen (Fig. 9b). Notably, we were unable to detect the intracellular localization of SlVPE3 and KTI4 fused to green fluorescent protein (GFP) or mCherry, which might be due to degradation and/or protonation of GFP or mCherry under the acidic conditions that are found in vacuoles of higher plants [47].

To confirm the colocalization of SlVPE3 and KTI4, their ORFs were fused to mRFP and PRpHluorin (plant-solubility-modified ratiometric pH-sensitive mutants of green fluorescent protein), respectively, and the fusion proteins were co-expressed in tobacco leaves. PRpHluorin, derived from GFP, is a fluorescent pH sensor that has been successfully used to characterize the localization of vacuolar proteins [48]. We observed that the fluorescent signals of mRFP co-localized with those of PRpHluorin, suggesting the subcelluar colocalization of SlVPE3 and KTI4 (Fig. 9c).

### SlVPE3 participates in the cleavage of KTI4

**Fig. 10:**
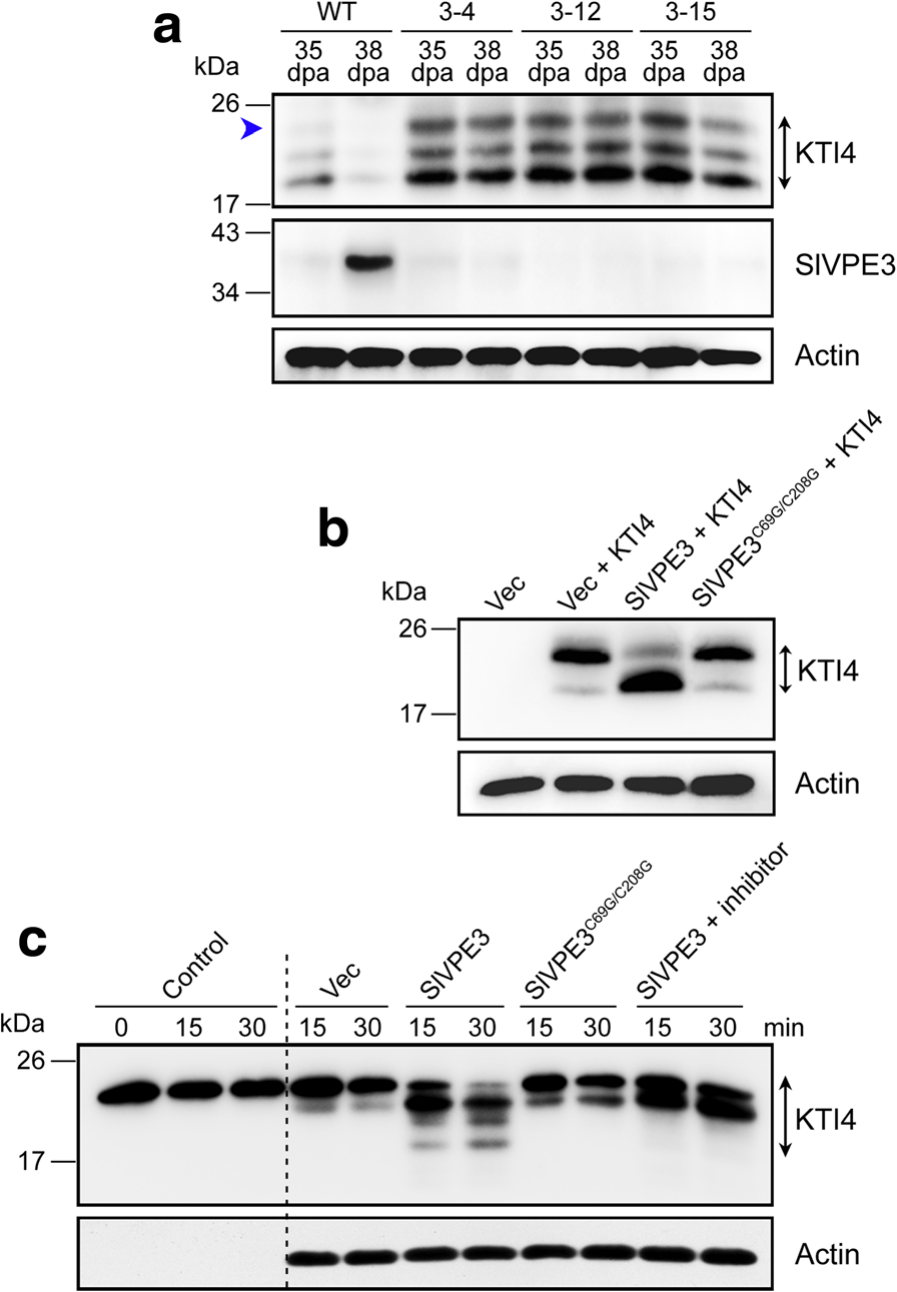
SlVPE3 is involved in KTI4 cleavage. **a** Immunoblot detection of KTI4 cleavage in tomato fruit. Total protein extracts from wild-type (WT) and *SlVPE3* RNAi fruit (3-4, 3-12, and 3-15) at 35 and 38 days post-anthesis (dpa) were analyzed by immunoblot analysis using anti-KTI4 or anti-SlVPE3 antibodies. The predicted molecular mass of the full-length KTI4 is ~25 kDa, which is indicated by a *blue arrowhead*. **b** Determination of KTI4 cleavage in tobacco (*N. benthamiana*). Proteins were extracted from tobacco leaves expressing an empty vector (*Vec*), KTI4 alone (*Vec + KTI4*), SlVPE3 and KTI4 (*SlVPE3 + KTI4*), and mutated SlVPE3 and KTI4 (*SlVPE3* ^*C69G/C208G*^ *+ KTI4*) and subjected to immunoblot analysis with an anti-KTI4 antibody. The mutated form of SlVPE3 (SlVPE3^C69G/C208G^) was generated by site-directed mutagenesis. **c** Cell-free cleavage of His-tagged KTI4 proteins. His-KTI4 was expressed and purified from *Escherichia coli* and then incubated for 15 or 30 min at 20 °C with extracts of *N. benthamiana* expressing an empty vector (*Vec*), intact SlVPE3 (*SlVPE3*), or a mutated form of SlVPE3 (*SlVPE3* ^*C69G/C208G*^). An incubation of His-KTI4 with extraction buffer served as the control. The VPE inhibitor (biotin-YVAD-fmk) was applied to determine whether the His-KTI4 was directly cleaved by SlVPE3. In **a**–**c**, equal loading was confirmed with an anti-actin antibody. Similar results were obtained from three independent experiments and results from a representative experiment are shown

Having demonstrated that SlVPE3 protein interacts and colocalizes with KTI4, we then raised polyclonal antibodies against KTI4 to test whether SlVPE3 participates in the processing of KTI4. Polyclonal antibodies were affinity-purified and immunoblot analysis indicated no immunoreactive bands in extracts from wild-type tomato fruit at 35 dpa when pre-immune serum was used (Additional file 4: Figure S4), but a signal was detected by the affinity purified anti-KTI4 antibodies corresponding to the size (25 kDa) of the predicted full-length KTI4 polypeptide (Fig. 10a, blue arrowhead). The affinity purified anti-KTI4 antibodies recognized two additional bands with lower molecular mass (Fig. 10a), suggesting that KTI4 undergoes cleavage to form smaller peptides. We observed that KTI4 protein levels were higher in fruit of the *SlVPE3*-silenced lines (3-4, 3-12, and 3-15) and in wild type when SlVPE3 levels were low, at 35 dpa (Fig. 10a). Conversely, the accumulation of KTI4 was reduced when SlVPE3 levels were high (Fig. 10a). These results are consistent with SlVPE3 being involved in KTI4 processing. Notably, the total abundance of KTI4 in *SlVPE3* RNAi fruit appeared to be higher than in the wild type, which is consistent with the iTRAQ analysis showing that KTI4 abundance increased significantly after *SlVPE3* was repressed. The changes in protein levels of KTI4 might reflect direct proteolytic processing of KTI4 by SlVPE3, or by the increase in KTI4 transcript levels (Fig. 6), or both.

To verify that SlVPE3 cleaves KTI4, the *SlVPE3* and *KTI4* ORFs were transiently expressed in tobacco (*N. benthamiana*) leaves. As shown in Fig. 10b, a band of the predicted molecular mass of the full-length KTI4 (~25 kDa) was generated by the anti-KTI4 antibodies, together with an additional faint band with a lower molecular mass. When *SlVPE3* was co-expressed with *KTI4* in tobacco, the abundance of the 25-kDa band decreased, concomitant with an increase in the intensity of the lower molecular mass band (Fig. 10b), suggesting that the 25-kDa band constitutes a precursor of the lower molecular mass band. Importantly, when *KTI4* was co-expressed with a mutated form of SlVPE3 (SlVPE3^C69G/C208G^) in which conserved cysteine (C) residues (C69 and C208) from the active site were replaced by glycine (G), the changes in abundance of the two bands were abolished (Fig. 10b), indicating that SlVPE3 must be catalytically active for KTI4 to be cleaved.

We subsequently examined the cleavage of KTI4 by SlVPE3 in vitro. The predicted mature SlVPE3 polypeptide and KTI4 without the signal peptide were independently expressed in *Escherichia coli* as fusion proteins with a His-tag. Substantial amounts of recombinant SlVPE3 protein were obtained but did not show VPE activity (data not shown). We also expressed SlVPE3 in *Saccharomyces cerevisiae* BY4741 and *Pichia pastoris* GS115 cells, but did not detect proteolytically active recombinant proteins (data not shown). We next investigated the cleavage of the His-tagged recombinant KTI4 proteins (His-KTI4) in cell-free extracts of tobacco expressing intact or mutated SlVPE3 (SlVPE3^C69G/C208G^). A 25-kDa band, in addition to a band with lower molecular mass, was detected by the anti-KTI4 antibodies when extracts of tobacco expressing the empty plasmid were incubated with His-KTI4 (Fig. 10c). Two additional bands were observed in extracts of tobacco expressing intact SlVPE3, suggesting the cleavage of KTI4 by SlVPE3 (Fig. 10c). Gradual processing of KTI4 was observed when the reaction time was prolonged. The processing could be blocked in extracts of tobacco expressing the mutated form of SlVPE3 (SlVPE3^C69G/C208G^), suggesting specific cleavage of KTI4 by SlVPE3. To determine whether KTI4 is directly cleaved by SlVPE3, biotin-YVAD-fmk [33], a VPE inhibitor that efficiently inhibits the activity of SlVPE3 (Additional file 4: Figure S5), was added to the reaction. Application of the inhibitor blocked KTI4 processing (Fig. 10c), further demonstrating that KTI4 is a direct target of SlVPE3.

### KTI4 does not inhibit the activity of SlVPE3

**Fig. 11:**
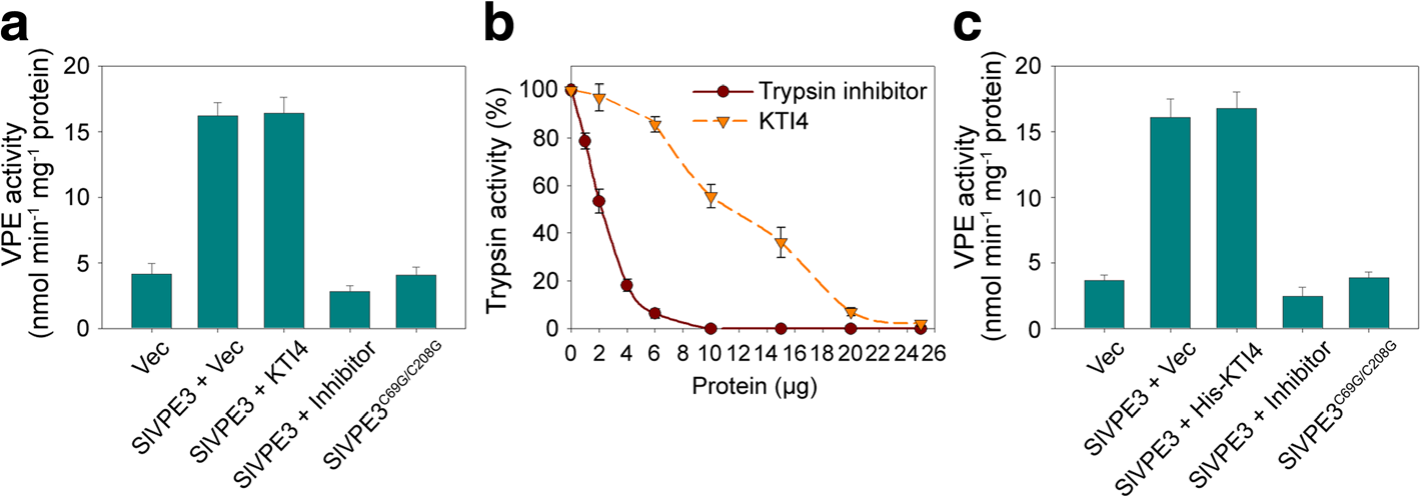
KTI4 does not inhibit SlVPE3 proteolytic activity. **a** Determination of vacuolar processing enzyme (*VPE*) activity in tobacco (*N. benthamiana*). Proteins were extracted from *N. benthamiana* leaves expressing an empty vector (*Vec*), SlVPE3 alone (*SlVPE3 + Vec*), SlVPE3 and KTI4 (*SlVPE3 + KTI4*), and mutated SlVPE3 (*SlVPE3* ^*C69G/C208G*^) and subjected to a VPE activity assay with a VPE-specific fluorescent substrate. The mutated form of SlVPE3 (SlVPE3^C69G/C208G^) was generated by site-directed mutagenesis. The VPE inhibitor biotin-YVAD-fmk was added. Values are shown as the means ± standard deviation (SD). **b** The recombinant KTI4 protein from *E. coli* showed inhibitory activity against trypsin. Soybean trypsin inhibitor was used as a positive control. Trypsin activity was measured with the Nα-benzoyl-L-arginine ethyl ester substrate. *Error bars* represent the SD of three independent experiments. **c** VPE activity assay with recombinant KTI4 protein (His-KTI4). Extracts from *N. benthamiana* expressing an empty vector (*Vec*), intact SlVPE3, or a mutated form of SlVPE3 (*SlVPE3* ^*C69G/C208G*^) were incubated with recombinant KTI4 protein purified from *E. coli*, and then subjected to a VPE activity assay. Values are shown as the means ± SD

As a protease inhibitor, KTI4 has the potential to block protease activity. KTIs are specific for serine proteases, and have specific inhibitory activity solely against trypsin proteases that cleave polypeptides after Lys or Arg [45]. Accordingly, it is unlikely that KTI4 inhibits the activity of VPE proteins, as they are cysteine proteases. However, to eliminate the possibility that KTI4 inhibits SlVPE3 activity, we transformed *KTI4* and *SlVPE3* into tobacco and measured VPE activity with a fluorescent substrate. A fluorescent signal (3.34 nmol min^−1^ mg^−1^ protein) representing VPE activity was detected in tobacco leaves transformed with an empty plasmid control (Fig. 11a), indicating that tobacco itself contains VPE activity. However, when tobacco was transformed with *SlVPE3*, higher VPE activity (4.7 times; 15.6 nmol min^−1^ mg^−1^ protein) was detected, while tobacco expressing the mutated form of SlVPE3 (SlVPE3^C69G/C208G^) showed similar VPE activity to the empty vector control. The VPE activity was inhibited by the specific inhibitor biotin-YVAD-fmk. However, no significant differences in VPE activity were found in tobacco leaves co-expressing *SlVPE3* and *KTI4* compared with those expressing *SlVPE3* alone (Fig. 11a), suggesting that KTI4 does not inhibit VPE activity.

We also evaluated the effects of recombinant KTI4 proteins purified from *E. coli* on VPE activity. The recombinant KTI4 proteins exhibited activity against trypsin (Fig. 11b), indicating that they were active serine protease inhibitors. The incubation of the KTI4 proteins with extracts from tobacco leaves expressing intact or mutated SlVPE3 (SlVPE3^C69G/C208G^) did not significantly affect VPE activity, although this activity was inhibited by the specific inhibitor biotin-YVAD-fmk (Fig. 11c).

### KTI4 contributes to resistance of fruit to pathogen infection

**Fig. 12:**
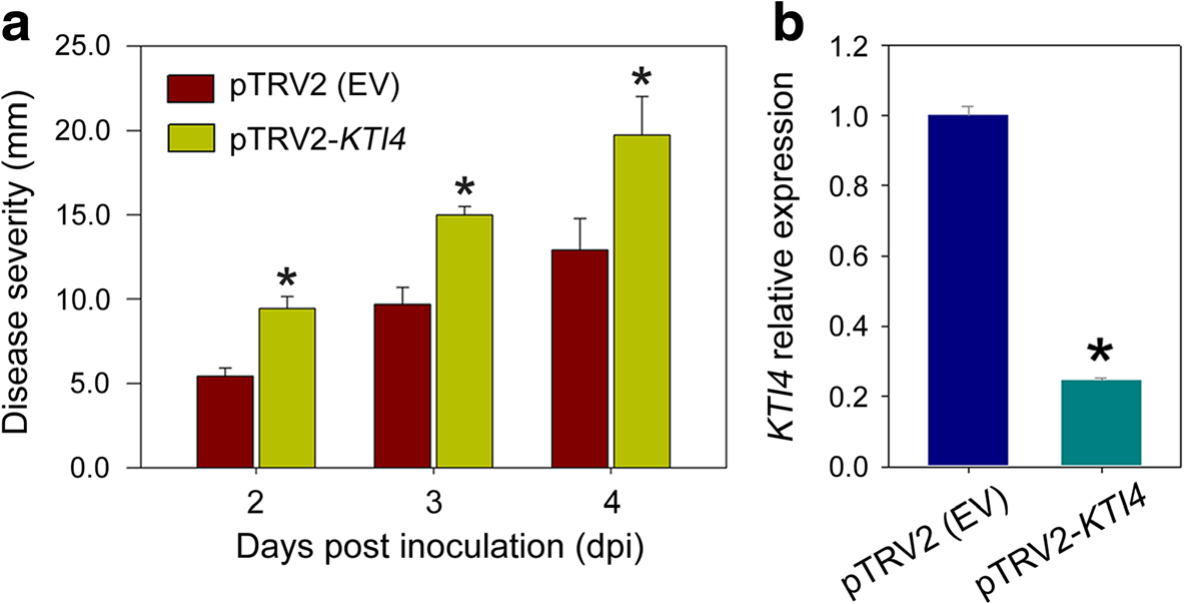
KTI4 influences disease resistance in tomato fruit. **a** Virus-induced gene silencing (VIGS) assay revealed that *KTI4* affects disease resistance in tomato fruit. Immature green stage fruit were infiltrated with an empty vector control pTRV2 (*EV*) or pTRV2 containing a specific *KTI4* sequence (pTRV2-*KTI4*). Fourteen days after infiltration, the fruit were inoculated with *B. cinerea*. Disease severity was observed daily after inoculation. **b** *KTI4* gene expression after VIGS, as determined by quantitative RT-PCR. The gene transcript levels were normalized against expression of the *ACTIN* gene, followed by normalization against the control. Values are shown as the means ± standard deviation. *Asterisks* indicate significant differences (*P* < 0.05; *t*-test) between fruit infiltrated with pTRV2 (EV) and pTRV2-*KTI4*

To examine whether KTI4 provides fruit with resistance to pathogen infection, we performed a virus-induced gene silencing (VIGS) assay [49, 50]. A *KTI4* cDNA fragment was inserted into the pTRV2 vector, which was subsequently infiltrated into tomato fruit at the immature green stage. Fruit infiltrated with pTRV2 alone (empty vector) were used as a control. Fourteen days after infiltration, the fruit were inoculated with *B. cinerea* and the symptoms were visually inspected daily. As shown in Fig. 12a, fruit infiltrated with pTRV2-*KTI4* showed increased disease severity compared with fruit infiltrated with pTRV2 alone (control). Gene expression analysis indicated that the *KTI4* mRNA levels were reduced by ~75% compared with the control (Fig. 12b).

### SlVPE3 is regulated by the RIN transcription factor

**Fig. 13:**
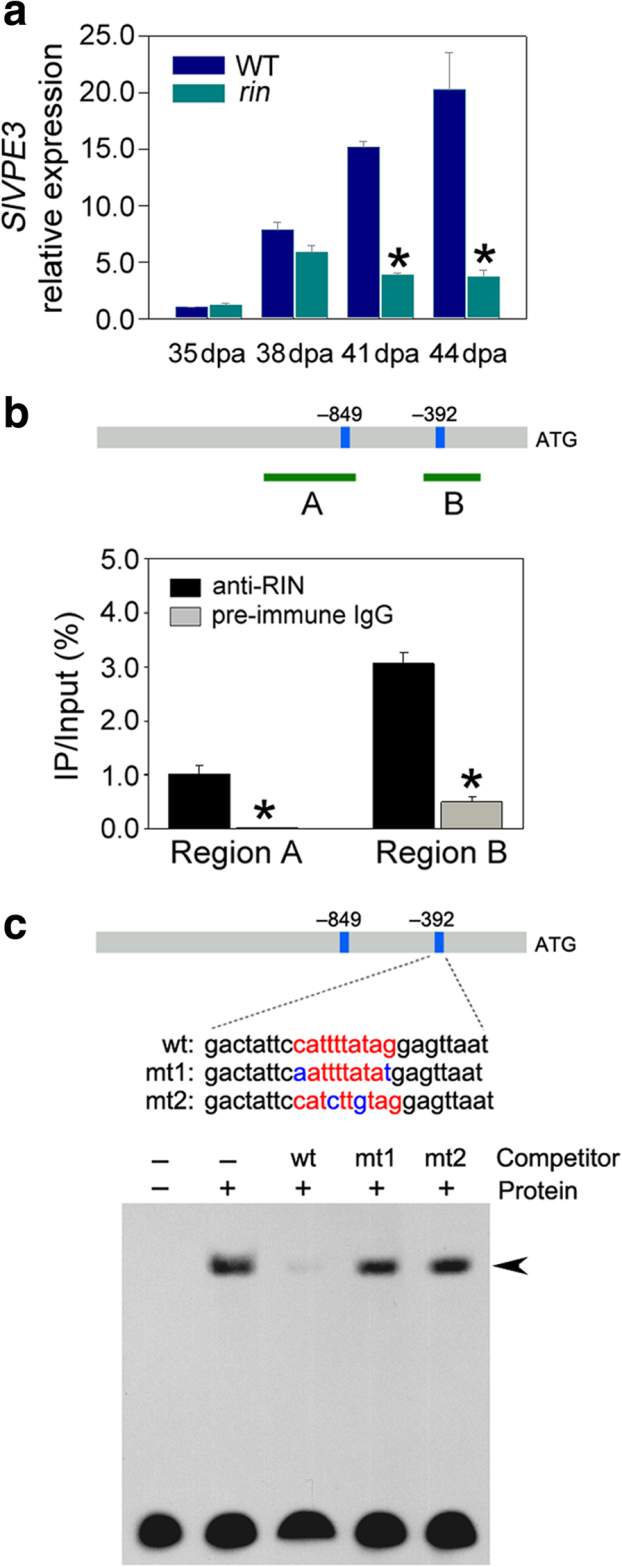
Regulation of *SlVPE3* by the RIN transcription factor. **a** *SlVPE3* expression in wild type (*WT*) and the *rin* mutant during fruit ripening, as determined by quantitative RT-PCR. The gene transcript levels were normalized against the *ACTIN* gene, followed by normalization against WT at 35 days post-anthesis (*dpa*). Values are shown as the means ± standard deviation (SD). *Asterisks* indicate significant differences (*P* < 0.05; *t*-test) between WT and the *rin* mutant. **b** ChIP-quantitative PCR assays indicated that RIN directly binds to the promoter of *SlVPE3*. The promoter structure of *SlVPE3* is shown. *Blue boxes* represent CArG box elements and *numbers* indicate the position of these motifs relative to the translational start site. *Green fragments with upper-case letters* represent the regions used for ChIP-quantitative PCR. Values are shown as the means ± SD. *Asterisks* indicate significant differences (*P* < 0.05; *t*-test) between samples co-immunoprecipitated with anti-RIN antibodies and pre-immune serum. **c** Gel mobility shift assays revealed the direct binding of RIN to the CArG box element in the promoter region of *SlVPE3*. The probe sequences corresponding to the *SlVPE3* promoter are shown, with *red letters* representing the CArG box. The mutated bases in the probes are represented by *blue letters. wt*, probe with intact CArG box element; *mt*, probe with mutated CArG box element. One thousand-fold excess amounts of unlabeled probes were added to the binding reaction as a competitor. The specific complexes formed are indicated by *arrowheads*

The activity of proteases needs to be tightly regulated due to their critical roles in diverse of biological processes. VPEs have been shown to be self-catalytically activated by sequential removal of C-terminal and N-terminal propeptides [51–53]. *SlVPE3* shows a similar expression pattern to the transcription factor RIPENING INHIBITOR (RIN), a global regulator of tomato fruit ripening [1, 9]. Quantitative RT-PCR analysis indicated that the expression of *SlVPE3* was decreased in fruit of the *rin* mutant tomato compared with wild type (Fig. 13a). To investigate whether RIN regulates the expression of *SlVPE3* by directly binding to its promoter in vivo, a ChIP assay was performed. Using PlantCARE [54], two CArG-box elements, which are typical binding sites for RIN [55], were identified in the promoter region of *SlVPE3* (Additional file 11: Table S10). For the ChIP assay, cross-linked DNA–protein complexes were immunoprecipitated with affinity-purified anti-RIN polyclonal antisera. The promoter sequences surrounding the CArG-box binding sites were amplified from the immunoprecipitated DNA using specific primers designed for *SlVPE3*, indicating that RIN binds to the promoter of *SlVPE3* in vivo (Fig. 13b). Binding of RIN to the promoter of *ACS2* was used as a positive control.

We also performed an electrophoretic mobility shift assay (EMSA) with the purified recombinant RIN protein. A band shift was observed when the purified RIN protein was mixed with the biotin-labeled probe (26-mer oligonucleotide) containing the CArG-box element. Binding of the RIN protein to the promoter fragment was out-competed by addition of an excessive amount of the corresponding unlabeled probe (competitor) containing an intact CArG box element, but not by the probe with a mutated CArG box element (Fig. 13c). This confirmed direct binding of RIN to the *SlVPE3* promoter.

## Discussion

In recent years, substantial progress has been made in understanding the transcriptional control of fruit ripening, but relatively little is known about the regulation of fruit ripening at the post-transcriptional level. In the present study, we show that SlVPE3, a cysteine protease, is involved in fruit ripening. Repression of *SlVPE3* resulted in a delay in ripening and made the fruit more susceptible to pathogen infection. Via a quantitative proteome study we found that SlVPE3 affects the abundance of a set of proteins associated with fruit ripening and disease resistance. Further analysis indicated that SlVPE3 participates in the cleavage of KTI4, a protease inhibitor that contributes to fruit disease resistance.

### SlVPE3 regulates fruit ripening at the post-transcriptional level

Proteases represent one of the most abundant classes of enzymes in eukaryotes and reside in various cellular compartments, such as the cytosol, mitochondria, vacuoles, lysosomes, endoplasmic reticulum, or the extracellular domain [23, 27, 56, 57]. In *A. thaliana*, more than 800 proteases have been identified, representing approximately 2% of the genome [27]. The distribution and the protease family size are well conserved within the plant kingdom [58]. However, even though they have been demonstrated to participate in a variety of biological processes [23, 27, 59–61], their roles in fruit ripening remain unclear. In this study, we showed that *SlVPE3*, a gene encoding a vacuolar processing enzyme, is required for normal tomato fruit ripening.

The iTRAQ-based quantitative proteome analysis represents a powerful technique for high-throughput nonbiased discovery of protease targets [62–64], and has provided increased coverage and sensitivity for quantitative proteomics [65]. Using this technique, more than 300 proteins that changed abundance upon *SlVPE3* silencing were identified, of which a set was associated with fruit ripening. These results suggested that *SlVPE3* regulates fruit ripening by affecting the accumulation of numerous ripening-related proteins. Notably, besides SlVPE3 targets, other proteins that changed abundance due to the delayed ripening were also identified. The proteins that differentially accumulated due to the activity of SlVPE3 itself might be identified by comparing the protein profiles between tomato leaves expressing an intact or mutagenized form of SlVPE3.

VPEs can degrade target proteins or activate substrate proteins from inactive precursor isoforms to active forms [23]. Interestingly, a homolog of SlVPE3, SlVPE5 (formerly named LeCp), was reported to function as a transcription factor and to regulate the expression of *ACS2* in tomato and tobacco (*N. tabacum*) leaves upon induction by the fungal elicitor EIX [42]. LeCp was found to bind directly to the *ACS2* promoter and it was proposed that LeCp functions as a protease in the cytoplasm and, upon elicitor signaling, enters the nucleus via binding by a small ubiquitin-related modifier (SUMO) protein, whereupon it acts as a transcription factor [42]. In our study, we found that SlVPE3 affects the transcript levels of several ripening-related genes (Fig. 6). However, ChIP-quantitative PCR analysis indicated that SlVPE3 does not bind to the promoters of these genes. In addition, we found no evidence for direct binding of SlVPE3 to the *ACS2* promoter. These results indicated that although LeCp, the homolog of SlVPE3, exhibits transcription factor activity that induces *ACS2* expression in tomato leaves, SlVPE3 does not act as a transcription factor in tomato fruit during ripening. Accordingly, SlVPE3 may regulate the ripening-related genes at the post-transcriptional levels rather than at the transcriptional levels. Alternatively, SlVPE3 may modulate the expression of ripening-related genes indirectly through processing of specific transcription factors, which in turn regulate the expression of these genes.

### Protease inhibitor KTI4 functions downstream of SlVPE3 to regulate fruit disease resistance

The susceptibility of tomato fruit to necrotrophic pathogens increases during fruit ripening [3, 30], and we hypothesized that since suppressing the expression of *SlVPE3* resulted in a delay in fruit ripening, it might also affect fruit susceptibility to pathogen infection. It has also been reported that simultaneously suppressing the expression of genes encoding the cell wall-modifying proteins polygalacturonase (*LePG*) and expansin (*LeExp1*) reduces the susceptibility of ripening tomato fruit to *B. cinerea* infection [3]. In this study, we found that a set of ripening-related proteins, including LePG and LeExp1, were less abundant in the *SlVPE3* RNAi fruit than in wild type. Contrary to expectations, however, we observed that the *SlVPE3* RNAi fruit were more susceptible to *B. cinerea* infection than the wild type (Fig. 3), suggesting that SlVPE3 may target proteins involved in defense responses, as well as those associated with ripening. Consistent with this idea, the proteomic data revealed that a number of proteins putatively associated with disease resistance have altered abundance in the *SlVPE3* RNAi fruit. In *A. thaliana*, knocking out the expression of *VPEγ* was also demonstrated to increase the susceptibility of leaves to pathogens [34], but the underlying molecular mechanisms are not well understood.

To identify proteins directly responsible for the SlVPE3-regulated disease resistance, we screened for proteins that interact with SlVPE3 using quantitative affinity purification followed by mass spectrometry, which detects protein–protein interactions in the native cellular environment and is suitable for detecting weak and transient interactors. A protease inhibitor, KTI4, was identified as a SlVPE3-interacting protein and the interaction was confirmed by a Y2H assay (Figs. 8 and 9). Immunoblot analysis of fruit from wild-type tomato and *SlVPE3* RNAi lines, and of tobacco leaves transiently expressing *SlVPE3* and *KTI4*, using antibodies against KTI4 showed that SlVPE3 participates in the cleavage of KTI4 (Fig. 10a, b). Furthermore, KTI cleavage by SlVPE3 was confirmed by cell-free assays using recombinant KTI4 proteins (Fig. 10c). These data suggest that KTI4 is a direct target of SlVPE3.

KTIs are single-chain polypeptides of ~20 kDa belonging to the serine protease inhibitor family [45, 46]. *A. thaliana* KTI1 has been reported to be associated with leaf PCD in plant–pathogen interactions [45]; however, the function of KTI proteins in fruit disease resistance or their regulation has not been determined. In the present study, we found that KTI4 was processed by SlVPE3 (Fig. 10) and played a role in defense response of tomato fruit to *B. cinerea* (Fig. 12). Fruit in which *KTI4* expression was suppressed by VIGS showed similar susceptibility to *B. cinerea* as those from the *SlVPE3* RNAi lines (Figs. 3 and 12). Importantly, we observed that KTI4 does not inhibit SlVPE3 protease activity (Fig. 11). These data suggest that KTI4 functions as a downstream effector of SlVPE3 to regulate fruit disease resistance. Notably, suppression of *KTI4* by VIGS did not affect fruit ripening (Additional file 4: Figure S6). The mechanism by which KTI4 affects pathogen infection remains unknown, but we speculate that it may target proteases that are secreted by fungal pathogens as virulence factors.

### SlVPE3 is regulated at multiple levels

Due to the central roles of proteases in many biological processes and the irreversible nature of proteolysis, the action of proteases must be tightly controlled to prevent improper cleavage of substrate molecules [25]. Protease activities are regulated at multiple levels. The regulation can occur at the transcriptional level, and at the protein level by activation of inactive zymogens, or by the binding of inhibitors and cofactors [24]. Many proteases are synthesized as inactive zymogens, which contain inhibitory prodomains that must be removed for the protease to become active [25]. The activation can be either autocatalytic or performed by other proteases. VPEs have been reported to be self-catalytically activated from inactive proproteins into active VPEs [51–53]. In contrast to the understanding of post-transcriptional regulation of VPEs activity, little is known about the regulation of VPEs at the transcriptional level.

An analysis of the *SlVPE3* promoter region revealed two CArG box elements, which are binding sites for the RIN transcription factor, suggesting that *SlVPE3* may be transcriptionally regulated by RIN. Gene expression analysis, combined with ChIP and EMSA assays, supported this hypothesis, and indicated that RIN regulates the expression of *SlVPE3* by directly binding to its promoter (Fig. 13). RIN has been reported to directly regulate genes involved in a wide variety of biological processes, such as lycopene accumulation, ethylene production, chlorophyll degradation, and aroma formation [66, 67], but little is known about the regulation of protein degradation by RIN. We previously demonstrated that genes encoding E2 ubiquitin-conjugating enzymes, the components of the ubiquitin-proteasome, serves as direct targets of RIN [28], suggesting that RIN might regulate ubiquitin-mediated proteolysis. In this study, we show that RIN directly regulates protein degradation by targeting a specific protease, *SlVPE3*, which represents a previously unreported RIN direct target in a study involving ChIP coupled with DNA microarray analysis [67]. Notably, the expression of *SlVPE3* was only partially downregulated in the *rin* mutant fruit, suggesting that additional transcriptional regulators are necessary for *SlVPE3* expression.

## Conclusions

Our findings provide new insights into understanding the gene regulatory networks and proteolytic mechanisms that contribute to ripening and disease resistance in fruit. Future studies will investigate the target proteases of KTI4 and uncover the molecular mechanisms by which KTI4 regulates defense response in tomato fruit.

## Methods

### Plant material

Seeds of wild-type tomato (*Solanum lycopersicum* cv. Ailsa Craig) and the ripening mutant *rin* in the cv. Ailsa Craig background were kindly provided by Dr. James J. Giovannoni (Boyce Thompson Institute for Plant Research, Cornell University, Ithaca, NY, USA). Plants were grown in a greenhouse under standard culture conditions, with a regular supply of fertilizer and supplementary lighting when required. Flowers were tagged at anthesis to accurately follow fruit ages through development. Fruit ripening stages used in wild type were mature green (MG), breaker (Br), orange (Or), and red ripe (RR), which were defined on basis of the color, size, shape, seed development, and the development of locular jelly in the fruit [68]. These ripening stages (MG, Br, Or and RR) were on average 35, 38, 41, and 44 dpa, respectively. *rin* mutant fruit or transgenic lines were collected at the equivalent ripening stages, as determined by the number of dpa. Immediately after harvesting, pericarp tissue was collected, frozen in liquid nitrogen, and stored at −80 °C until use.

### RNA isolation and quantitative RT-PCR analysis

RNA was isolated from the pericarp using the method of Moore et al. [69]. The extracted RNA was treated with DNase I (Promega) and reverse transcribed using an oligo (dT)_18_ primer with Moloney murine leukemia virus (M-MLV) reverse transcriptase (Promega) to synthesize cDNA. Quantitative RT-PCR was carried out with the SYBR Green PCR Master Mix (Applied Biosystems) using the StepOne Plus Real-Time PCR System (Applied Biosystems). Gene-specific primers (Additional file 12: Table S11) were designed with the help of the Primer Express software 3.0 (Applied Biosystems). The following program was applied for PCR amplification in a volume of 20 μL: 95 °C for 10 min, followed by 40 cycles of 95 °C for 15 s and 60 °C for 30 s. Relative quantification of specific mRNA levels was performed using the cycle threshold (Ct) 2^(−ΔCt)^ method [70]. Expression values were normalized using *ACTIN* (SGN-U580609). Each experiment had three biological repeats, each with three technical replicates.

### Phylogenetic analysis

An alignment of the VPE protein sequences from tomato (*S. lycopersicum*) (Additional file 4: Supplementary text), rice (*Oryza sativa*), potato (*Solanum tuberosum*), tobacco (*N. tabacum*), pepper (*Capsicum annuum*), maize (*Zea mays*), soybean (*Glycine max*), citrus (*C. sinensis*), grape (*Vitis vinifera*) and *A. thaliana* was generated using ClustalX (version 2.1) software [71] with default multiple parameters and the PAM series protein weight matrix. The genedoc program was used to manually edit the alignment, which was then imported into MEGA (version 5.2) software [72] and the phylogenetic tree was constructed by the neighbor-joining statistical method using 1000 bootstrap replicates [72].

### Construction of the RNAi vector and plant transformation

To construct the *SlVPE3* RNAi plasmid, a 278-bp *SlVPE3* fragment was amplified from cDNA of tomato fruit at 38 dpa with *SlVPE3*-specific primers (*SlVPE3* RNAi/F: 5′-GTTCCCTCCACAGGGGTT-3′; *SlVPE3* RNAi/R: 5′-AGATGAAAGAAAGTTTGTTCAGG-3′) and cloned into the pCR8/GW/TOPO Gateway entry vector (Invitrogen). The cloned fragment was subsequently transferred into the binary RNAi vector pK7GWIWG2D [73]. The resulting construct was sequence confirmed and transformed into *A. tumefaciens* strain GV3101 [74], which was subsequently transformed into tomato (*S. lycopersicum* cv. Ailsa Craig) according to the method of Fillatti et al. [75]. The presence of the transgene was verified by PCR in the T0 and T1 tomato generations.

### Lycopene measurements

Pericarp lycopene content was measured as described by Sun et al. [76] and expressed as mg kg^−1^ fresh weight. Each sample contained three replicates with five fruit per replicate and the experiment was repeated twice.

### Ethylene production assay and ethephon treatment

To measure ethylene biosynthesis, fruit from transgenic T1 lines and the wild-type tomato were harvested at 38 and 41 dpa and placed in open jars for 3 h to avoid measuring “wound ethylene”, transiently synthesized as a consequence of picking. The jars were then sealed, incubated at room temperate for 2 h and then 1 mL gas samples were taken and analyzed by a gas chromatograph (SQ-206, Beijing, China) equipped with an activated alumina column and a flame ionization detector. Ethylene concentrations were calculated by comparing the peak length from the gas chromatograph with reagent grade ethylene standards of known concentration and normalizing for fruit weight. Each sample contained three replicates with five fruit per replicate and the experiment was repeated twice.

For the ethephon treatment, tomato fruit at 35 dpa were immersed for 10 min in a 50 mM fresh aqueous solution of ethephon (Sigma) or in water for the control. Pericarp samples were collected at various time intervals.

### Protein extraction, iTRAQ labeling, and NanoLC–MS/MS analysis

Proteins were extracted from tomato fruit pericarp at 41 and 44 dpa as previously described [77]. The isolated proteins were solubilized in protein buffer consisting of 500 mM triethylammonium bicarbonate (TEAB) and 1% SDS (w/v), pH 8.5, and the protein concentrations were determined by the Bradford method [78]. One-hundred micrograms of protein from each sample were reduced, alkylated, and digested using the filter-aided sample preparation (FASP) method [79]. The tryptic peptides were then labeled with the iTRAQ Reagents 4-plex Kit (Applied Biosystems) following the manufacturer’s protocol. Samples taken from wild-type and *SlVPE3* RNAi fruit at 41 dpa were labeled with iTRAQ tags 114 and 115, respectively, while samples from wild-type and *SlVPE3* RNAi fruit at 44 dpa were labeled with iTRAQ tags 116 and 117, respectively. The iTRAQ experiment was performed with two independent biological replicates. The iTRAQ-labeled samples were combined and subjected to high-pH reversed-phase chromatography. Briefly, the pooled iTRAQ-labeled peptides were reconstituted with buffer A (20 mM ammonium formate, pH 10, in water) and loaded onto a 4.6 × 250 mm, 150 Å size Durashell C18 (L) column containing 5 μm particles (Agela Technologies). The peptides were eluted at a flow rate of 0.8 mL min^−1^ using a gradient of 2% buffer B (20 mM ammonium formate in 80% acetonitrile, pH 10) for 5 min, 2–30% buffer B for 25 min, and 30–90% buffer B for 10 min. The system was then maintained in 90% buffer B for 10 min before equilibration with 2% buffer B for 10 min. The elution was monitored by measuring UV absorbance at 210 nm, and fractions were collected every 1 min. Forty-eight fractions were collected and pooled into a total of six fractions. After reconstitution in 0.1% formic acid, 8 μL of the combined iTRAQ-labeled peptides were submitted for NanoLC–MS/MS analysis.

The mass spectroscopy analysis was performed as previously described [80] using a NanoLC system (NanoLC–2D Ultra Plus, Eksigent) equipped with a Triple TOF 5600 Plus mass spectrometer (AB SCIEX). Protein identification and quantification for the iTRAQ experiments were performed using ProteinPilot™ 4.5 software (AB SCIEX). The mass spectra data were used to search the *S. lycopersicum* protein database ITAG2.4_proteins_full_desc.fasta, using the following parameters: (i) Sample type, iTRAQ 4-plex (Peptide Labeled); (ii) Cysteine alkylation, MMTS; (iii) Digestion, Trypsin; (iv) Instrument, TripleTOF 5600; (v) Species, *None*; (vi) Quantitate, Yes; (vii) Bias correction, Yes; (viii) Background correction, Yes; (ix) Search effort, Thorough; (x) FDR analysis, Yes. For iTRAQ quantification, the peptide for quantification was automatically selected using the Pro Group^TM^ algorithm (AB SCIEX) to calculate the reporter peak area. A reverse database search strategy [81] was used to estimate the global FDR for peptide identification. Only proteins identified below the 1% global FDR were ultimately exported for determining the meaningful cut-off value for the regulated proteins [82]. Hierarchical clustering (Pearson algorithm) was carried out with PermutMatrix software [31].

### Preparation of polyclonal antibodies

For SlVPE3-specific antibody preparation, a truncated form of *SlVPE3* lacking the conserved domain was amplified from tomato cDNA using primers F (5′-AGGATATCGAAAGACACAACCTGCG-3′) and R (5′-ACGTCGACCTAACCAGCAGGGCGGAC-3′) and inserted into the pET-30a vector (Merck KGaA). The resulting plasmid was transformed into *E. coli* BL 21 (DE3) competent cells. For recombinant protein expression, *E. coli* was cultured overnight and then diluted 1:100 in Luria Broth medium. The bacteria were incubated at 37 °C until A_600_ reached approximately 0.5, then isopropyl-1-thio-*β*-D-galactopyranoside (IPTG) was added to a final concentration of 1 mM to induce recombinant protein expression. The bacterial cells were incubated for an additional 3 h before the cells were collected by centrifugation for recombinant protein isolation. The recombinant protein was purified using Ni-NTA His Bind Resin according to the manufacturer’s manual (Merck KGaA), followed by further purification by 12% SDS-PAGE. The protein band corresponding to the predicted size of the recombinant SlVPE3 was excised from the gel and used to immunize rabbits at the Beijing Protein Institute Co., Ltd. Polyclonal antibodies that recognized SlVPE3 was affinity-purified from antisera using the AminoLink Plus Coupling Resin following the purification protocol (Thermo Scientific).

Polyclonal KTI4 antibodies were raised by injecting a rabbit with the synthetic peptide TYASVVDSDGNPVKAGAKYF followed by affinity-purification using the synthetic peptide.

### Immunoprecipitation of SlVPE3-interacting proteins for SWATH-MS analysis

Proteins were isolated from 41 dpa tomato fruit using IP buffer containing 50 mM Tris-HCl, pH 7.5, 150 mM NaCl, 1% NP-40, 50 μM MG132, 1 mM PMSF, and the protease inhibitor cocktail tablet (Roche). After centrifugation at 12,000 × *g* for 10 min, the supernatant containing the proteins was immunoprecipitated overnight at 4 °C with 50 μg of anti-SlVPE3 or pre-immune serum IgG (negative control) that was coupled to an agarose support, as described in the Pierce® Co-Immunoprecipitation (Co-IP) Kit (Pierce Biotechnology). The agarose beads were collected in spin columns and washed twice with IP buffer. The proteins were then eluted from the beads with 0.1 M glycine-HCl (pH 2.2), followed by reduction, alkylation, and digestion using the filter-aided sample preparation (FASP) method [79]. The resulting peptides were collected, dried under vacuum, and redissolved in 0.1% formic acid for NanoLC-MS/MS analysis. Quantitative analysis of SlVPE3-interacting proteins was performed using the SWATH-MS method [28]. Mass spectra were generated on a TripleTOF 5600 plus instrument (AB SCIEX) operating in the SWATH mode. Each sample contained three technical replicates and a *t*-test was used for statistical analysis. A *P* value <0.01 was considered to be significant.

### Y2H analysis

Construction and two-hybrid screening of the tomato cDNA library was performed with the Matchmaker Library Construction and Screening Kit (Clontech). Total RNA was extracted from 38 dpa (Br stage) tomato fruit pericarp and mRNA was isolated and used for cDNA library construction. The tomato cDNA library constructed in the prey vector pGADT7 (AD) was screened with a *SlVPE3* cDNA fragment encoding the mature protein cloned into the bait vector pGBKT7 (BD) in *S. cerevisiae* strain AH109 (Clontech), and positive clones were selected on SD/-Leu/-Trp/-His/-Ade medium supplemented with X-α-Gal.

To confirm the interactions between SlVPE3 and KTI4, the *SlVPE3* cDNA fragment encoding the mature protein and the ORF of *KTI4* were cloned into the AD and BD vectors, respectively, resulting in the SlVPE3-AD and KTI4-BD plasmids. Primers used for the vector construction are shown in Additional file 13: Table S12. The vectors were co-transformed into *S. cerevisiae* strain AH109 following the manufacturer’s manual (Clontech), and dripped on SD/-Leu/-Trp medium (SD/-2) and SD/-Leu/-Trp/-His/-Ade medium (SD/-4) containing X-α-Gal. As controls, AD and BD, KTI4-BD and AD, or SlVPE3-AD and BD were co-transformed. The experiments were repeated three times.

### Subcellular colocalization

For subcellular localization analysis, the full-length *SlVPE3* and *KTI4* cDNAs were amplified by PCR (primer sequences in Additional file 13: Table S12) and individually cloned into the pCambia 2300-MCS-mRFP vector. The resulting plasmids were transformed into *A. tumefaciens* GV3101 [74], which was subsequently infiltrated into tobacco (*N. benthamiana*) leaves [83]. For colocalization analysis, *N. benthamiana* plants co-expressing mRFP-tagged SlVPE3 (SlVPE3-mRFP) and PRpHluorin-tagged KTI4 (KTI4-PRpHluorin) under the control of the CaMV 35S promoter were generated. The plasmid containing *PRpHluorin* was kindly provided by Dr. Liwen Jiang (School of Life Sciences, The Chinese University of Hong Kong, Shatin, New Territories, Hong Kong, China). The tobacco plants were kept in the greenhouse for two days, and then mesophyll protoplasts were isolated [84] and observed under a Leica confocal microscope (Leica DMI600CS).

### Protein cleavage assay

Proteins from the pericarp of wild-type and *SlVPE3* RNAi fruit at 35 and 38 dpa were extracted using a phenol extraction method [77]. The proteins were solubilized in lysis buffer consisting of 7 M urea, 2 M thiourea, 4% CHAPS, and 1% dithiothreitol (DTT). Protein concentrations were determined by the Bradford method [78]. The samples were separated by 12% SDS-PAGE and subjected to immunoblot analysis, as below.

For the KTI4 cleavage assay in tobacco (*N. benthamiana*), the full-length *SlVPE3* and *KTI4* were amplified using gene-specific primers (Additional file 13: Table S12) and cloned into the pCambia 2300 vector (Cambia) to generate the 35S:SlVPE3 and 35S:KTI4 constructs. A mutated form of SlVPE3 (SlVPE3^C69G/C208G^) was constructed by site-directed mutagenesis using the QuikChange II XL site-directed mutagenesis kit (Agilent Technologies) according to the manufacturer’s instructions (primers are listed in Additional file 14: Table S13). The constructs were introduced into *A. tumefaciens* GV3101 [74] and then expressed transiently in *N. benthamiana* leaves [83] as described above. After 2 days, total proteins were extracted with an extraction buffer containing 25 mM Tris · HCl (pH 7.5), 150 mM NaCl, 1 mM EDTA, 1 mM DTT, 1% (v/v) TritonX-100, 1 mM PMSF, and a protease inhibitor cocktail tablet (Roche). The homogenates were centrifuged at 12,000 × *g* for 10 min at 4 °C, and then the supernatants were subjected to immunoblot analysis, as below.

### Cell-free assays for protein cleavage

The cell-free cleavage assay was carried out as previously described [85]. The *KTI4* coding sequence without the predicted signal peptide region was amplified from cDNA of tomato fruit using the primers KTI4-F (5′-GGGGTACCATGTCAACATTTTCTTCAGATCTT-3′) and KTI4-R (5′-GCGTCGACttaATCAGCCTTCTTGAAGTAA-3′) and cloned into the pCold I vector (Takara) to produce pCold I-*KTI4*. This construct allows an in-frame fusion of the coding region of *KTI4* with an N-terminal His-tag. The plasmid was transformed into *E. coli* BL 21 (DE3) cells, and the recombinant protein expression and purification were performed as described above. For the cleavage assay, total proteins were extracted from *N. benthamiana* leaves that transiently expressed the intact or mutated form of SlVPE3 (SlVPE3^C69G/C208G^) using an extraction buffer containing 100 mM sodium acetate, pH 5.5, 1 mM PMSF, 100 mM DTT, and 100 μm E64-d. After two rounds of centrifugation at 12,000 × *g* for 10 min each at 4 °C, the supernatant was collected and the protein concentration was determined using the Bradford method [78]. The total protein extracts (250 μg) were incubated with 200 ng of purified recombinant His-KTI4 in 250 μL extraction buffer for 0, 15, and 30 min at 20 °C. Wherever indicated, the VPE inhibitor biotin-YVAD-fmk was used at 100 μM by pre-incubation with the total protein extracts for 30 min before incubation with His-KTI4. The mixtures were subjected to SDS-PAGE followed by immunoblot analysis, as below.

### Immunoblot analysis

For immunoblot analysis, proteins were separated by 12% SDS-PAGE and electrotransferred to an Immobilon-P PVDF membrane (Millipore). The membranes were blocked for 2 h at room temperature with 5% BSA in a PBS-Tween buffer (137 mM NaCl, 2.7 mM KCl, 8.1 mM NaH_2_PO_4_, 1.5 mM KH_2_PO_4_ and 0.1% Tween-20). Immunoblotting was conducted at 4 °C overnight with anti-KTI4 (1:1000) or anti-SlVPE3 (1:1000) antibodies. The membranes were washed with PBS-Tween (3 × 10 min), and then the secondary antibodies conjugated to horseradish peroxidase (Abmart) were added (1:5000). Immunoreactive bands were visualized using a chemiluminescence detection kit (SuperSignal®, Pierce Biotechnology). Equal loading was confirmed with an anti-actin antibody (Abmart).

### VPE activity assay

VPE activity was measured as previously described [86] using a synthesized fluorescent VPE-specific substrate, Ac-ESEN-MCA [Acetyl-Glutamyl-Seryl-Glutamyl-Asparagine α-(4-Metyl-Coumaryl-7-Amide)] (Shanghai Bootech BioScience & Technology Co., Ltd). Proteins were extracted with a buffer containing 100 mM sodium acetate, pH 5.5, 1 mM PMSF, 100 mM DTT, and 100 μm E64-d from leaves of *N. benthamiana* that transiently expressed intact SlVPE3 (alone or co-expressed with KTI4) or the mutated form of SlPVE3 (SlVPE3^C69G/C208G^) for 2 days. The samples were centrifuged at 12,000 × *g* for 15 min and the supernatants were used for VPE activity assays. The crude enzyme extract was incubated with 100 μM Ac-ESEN-MCA in an acidic buffer containing 100 mM sodium-acetate (pH 5.5) and 100 mM DTT for 2 h at 20 °C. The fluorescence intensity was measured using a Synergy™ H4 Multimode Microplate Reader (Bio-Tek Instruments, Inc.) with an excitation wavelength at 380 nm and an emission wavelength at 460 nm. VPE inhibitor biotin-YVAD-fmk was added to a final concentration of 100 μM by pre-incubating with the crude enzyme for 30 min before Ac-ESEN-MCA addition, wherever indicated.

The trypsin inhibitor activity of recombinant KTI4 was determined as previously described [45]. Trypsin (1 μg/μL, sigma) was pre-incubated with increasing concentrations of KTI4 in the assay buffer [45] for 30 min at 30 °C. The trypsin substrate N-benzoyl-L-arginine ethyl ester (Sigma) was then added to a final concentration of 0.25 mM and the changes in absorbance at 253 nm were monitored. Soybean trypsin inhibitor (Sigma) was used as a positive control. For the VPE activity assay with His-tagged recombinant KTI4 proteins, the purified KTI4 proteins (20 μg) were pre-incubated for 30 min with crude enzyme extracts from leaves of *N. benthamiana* that transiently expressed intact or the mutated form of SlPVE3 (SlVPE3^C69G/C208G^). Ac-ESEN-MCA was then added, as described above.

### Virus-induced gene silencing

The VIGS assay was performed as previously described [87, 88]. The virus vectors pTRV1 and pTRV2 were provided by Dr. Daqi Fu (College of Food Science and Nutritional Engineering, China Agricultural University, Beijing, China). The specific *KTI4* cDNA fragment was amplified using the primers F (5′-GGAATTCATGATGAAGAGCCTTGTTC-3′) and R (5′-CCGCTCGAGAGGACGTCCAGTGTTAAGTTCC-3′) and inserted into the pMD19-T vector (TaKaRa Bio). The resulting plasmid was transformed into *E. coli* and the sequence was verified. The cDNA fragment was subsequently cloned into the virus vector pTRV2 and then transferred to *A. tumefaciens* strain GV3101. For infiltration, equivalent aliquots of *Agrobacterium* strain GV3101 (with an optical density of 0.15 at 600 nm) containing pTRV1 or pTRV2 (empty or containing the insert) were mixed and injected into immature green stage tomato fruit. Agroinjected fruit were stored at room temperature (20 °C) for 14 days before infection with *B. cinerea*.

### Microbial infection

Infection of tomato fruit by *B. cinerea* was carried out as previously described [89]. *B. cinerea* (1 × 10^5^ conidia per ml) was applied to three sites that were equally spaced across the fruit surface. There were three replicates for each sample with at least ten fruit per replicate, and the experiment was repeated twice. Fungal growth was determined using quantitative PCR amplification of *B. cinerea ACTIN 2* relative to the tomato *ACTIN* gene as described by Laluk and Mengiste [90]. The following primer pairs were used for quantitative PCR: *B. cinerea ACTIN 2* forward (5′-ACTCATATGTTGGAGATGAAGCGCA-3′), reverse (5′-AATGTTACCATACAAATCCTTACGGA-3′); tomato *ACTIN* forward (5′-ACAACTTTCCAACAAGGGAAGAT-3′) and reverse (5′-TGTATGTTGCTATTCAGGCTGTG-3′).

### ChIP and EMSA

For the ChIP assay, pericarps of fruit at 41 dpa were excised, fixed in 1% formaldehyde under a vacuum for 20 min, then powdered in liquid nitrogen, and chromatin complexes were isolated and sonicated as previously described [28]. The sonicated chromatin complexes were incubated with affinity purified polyclonal anti-SlVPE3/anti-RIN antibodies or pre-immune serum IgG (negative control) as previously described [28]. The cross-linking was then reversed, and the amount of each precipitated DNA fragment was determined by real-time PCR using specific primers (Additional file 8: Table S7). Values are expressed as the percentage of DNA fragments that co-immunoprecipitated with specific (anti-RIN) [66] or non-specific (IgG) antibodies relative to the input DNA.

For the EMSA, recombinant His-tagged RIN protein was prepared as previously described [28], and purified using Ni-NTA His Bind Resin according to the manufacturer’s instructions (Merck KGaA). The ability of RIN to bind to biotin-labeled oligonucleotide probes was determined with the Lightshift Chemiluminescent EMSA kit (Thermo Scientific), as previously described [28].

### Data access

The mass spectrometry data have been deposited to the ProteomeXchange Consortium [91] via the PRIDE partner repository [92] with the dataset identifier PXD002980 (http://www.ebi.ac.uk/pride).

## Additional files

Additional file 1: Table S1. Tomato cysteine proteinase information. (XLS 51 kb)
Additional file 2: Table S2. Expression profiles of tomato genes encoding cysteine proteases during fruit ripening. (XLS 54 kb)
Additional file 3: Table S3. Tomato vacuolar processing enzymes (VPEs) information. (XLS 15 kb)
Additional file 4: Supplementary figures S1–S6 and Supplementary text. **Figure S1.** Protein sequence alignment of tomato vacuolar processing enzymes (VPEs) using Clustal X. **Figure S2.** Prediction of the potential off-targets of the RNAi construct. **Figure S3.** Identification of SlVPE3 by mass spectrometry. **Figure S4.** Determination of KTI4 antibody specificity. **Figure S5.** Effects of biotin-YVAD-fmk on vacuolar processing enzyme (VPE) activity in tobacco (*N. benthamiana*) leaves transiently expressing *SlVPE3*. **Figure S6.** Virus-induced gene silencing (VIGS) of *KTI4* in tomato has no effect on fruit ripening. Supplementary text Protein sequences of tomato vacuolar processing enzymes (VPEs). (PDF 1450 kb)
Additional file 5: Table S4. Similarities in the amino acid sequence of tomato vacuolar processing enzymes (VPEs). (XLS 16 kb)
Additional file 6: Table S5. Identification of differentially expressed proteins in the *SlVPE3* RNAi tomato fruit using iTRAQ-based quantitative proteomic analysis. (XLS 73 kb)
Additional file 7: Table S6. Predicted vacuolar processing enzyme (VPE) binding motifs within the 2000-bp upstream region starting from ATG. (XLS 16 kb)
Additional file 8: Table S7. Primers used in the ChIP-quantitative PCR analysis. (XLS 16 kb)
Additional file 9: Table S8. Identification of proteins that interact with SlVPE3 using SWATH-MS. (XLS 168 kb)
Additional file 10: Table S9. Proteins interacting with SlVPE3 (bait) in a yeast two-hybrid screen against a tomato prey library. (XLS 19 kb)
Additional file 11: Table S10. Predicted RIN binding motifs within a 2000-bp upstream region starting from start ATG of the *SlVPE3* gene coding sequence. (XLS 14 kb)
Additional file 12Table S11. Primers used for quantitative RT-PCR analysis. (XLS 47 kb)
Additional file 13: Table S12. Primers used in the yeast two-hybrid (Y2H) analysis, subcellular colocalization of SlVPE3 and KTI4, and tobacco (*N. benthamiana*) expression of *SlVPE3* and *KTI4*. (XLS 15 kb)
Additional file 14: Table S13. Primers used for *SlVPE3* mutagenesis. (XLS 13 kb)

## Abbreviations

AD: Activation domain
BD: Binding domain
Br: Breaker
ChIP: Chromatin immunoprecipitation
dpa: Days post-anthesis
DTT: Dithiothreitol
EMSA: Electrophoretic mobility shift assay
FDR: False discovery rate
GFP: Green fluorescent protein
iTRAQ: Isobaric tags for relative and absolute quantification
MG: Mature green
mRFP: Monomeric red fluorescent protein
MS: Mass spectroscopy
Or: Orange
ORF: Open reading frame
PCD: Programmed cell death
RNAi: RNA interference
RR: Red ripe
RT-PCR: reverse transcription polymerase chain reaction
SWATH-MS: Sequential window acquisition of all theoretical mass spectra
VIGS: virus-induced gene silencing
VPE: Vacuolar processing enzyme
Y2H: Yeast two-hybrid
