# Supplementary material for: Post-transcriptional regulation of fruit ripening and disease resistance in tomato by the vacuolar protease SlVPE3
Source: Genome Biol. 2017 Mar 7;18:47. doi: 10.1186/s13059-017-1178-2 (PMC5341188; doi:10.1186/s13059-017-1178-2)
Supplement: Additional file 4: — Supplementary figures S1–S6 and Supplementary text. Figure S1. Protein sequence alignment of tomato vacuolar processing enzymes (VPEs) using Clustal X. Figure S2. Prediction of the potential off-targets of the RNAi construct. Figure S3. Identification of SlVPE3 by mass spectrometry. Figure S4. Determination of KTI4 antibody specificity. Figure S5. Effects of biotin-YVAD-fmk on vacuolar processing enzyme (VPE) activity in tobacco (N. benthamiana) leaves transiently expressing SlVPE3. Figure S6. Virus-induced gene silencing (VIGS) of KTI4 in tomato has no effect on fruit ripening. Supplementary text Protein sequences of tomato vacuolar processing enzymes (VPEs). (PDF 1450 kb) [file 13059_2017_1178_MOESM4_ESM.pdf]

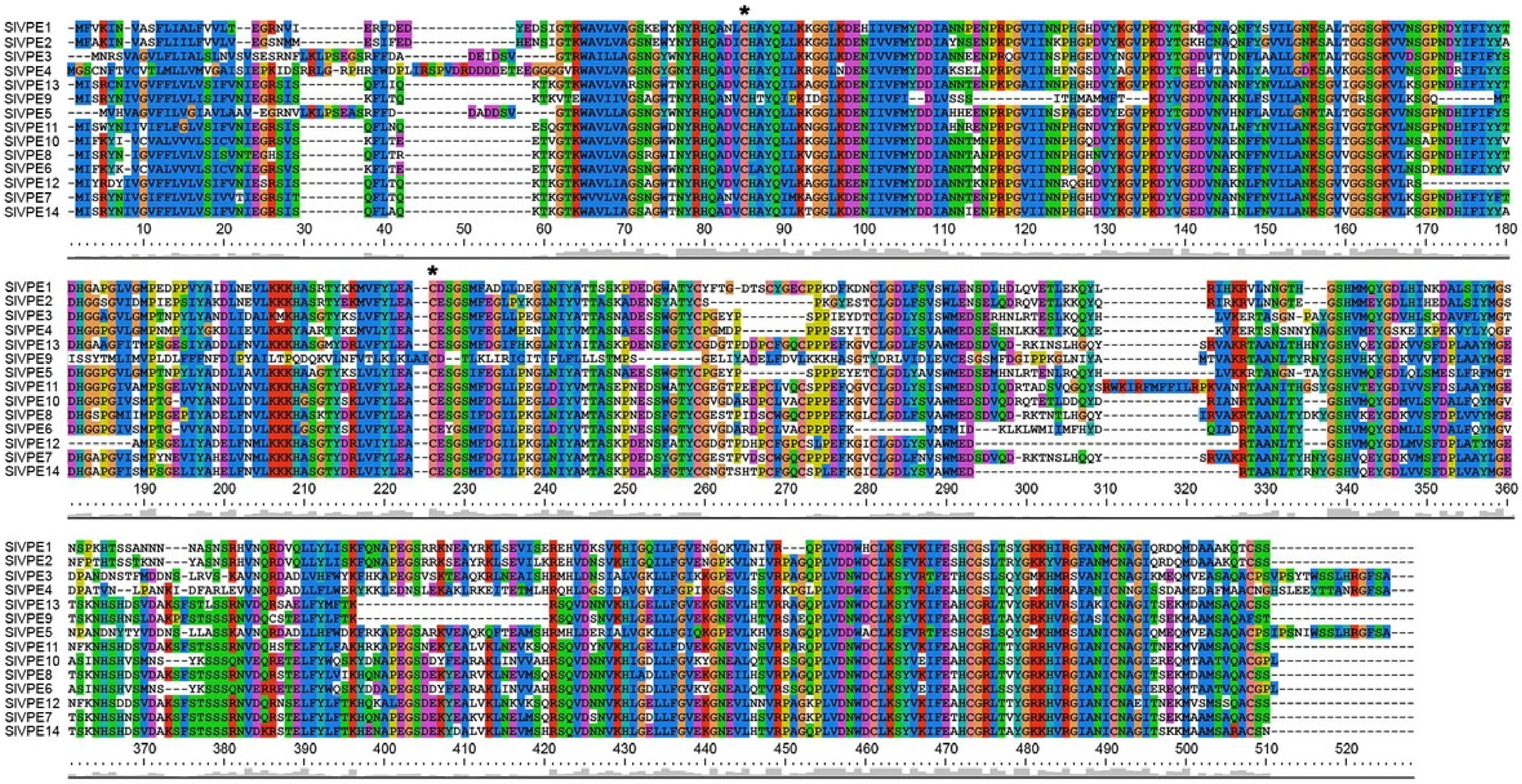

Additional file 4: Figure S1. Protein sequence alignment of tomato vacuolar processing enzymes (VPEs) using Clustal X. The amino acid sequences of all tomato VPEs were used to generate the alignment. Asterisks indicate conserved cysteine residues in the active-sites.

(a)

Number	Locus	Description
1	Solyc01g007850	Pentatricopeptide repeat-containing protein
2	Solyc01g099000	Pentatricopeptide repeat-containing protein
3	Solyc01g103620	Sodium bile acid symporter family protein expressed
4	Solyc01g106230	B3 domain-containing protein At5g60142
5	Solyc02g069490	FAD linked oxidase domain protein
6	Solyc03g071560	RNA recognition motif, glycine rich protein
7	Solyc03g077890	Unknown protein
8	Solyc03g082990	Tetratricopeptide repeat containing protein
9	Solyc04g056630	CBS domain containing protein-like
10	Solyc06g005670	mRNA binding protein Pumilio 2
11	Solyc07g049550	1-aminocyclopropane-1-carboxylate oxidase
12	Solyc08g079050	Unknown Protein (AHRD V1)
13	Solyc08g082900	WD-40 repeat family protein
14	Solyc09g065010	mRNA-capping enzyme subunit alpha
15	Solyc10g081920	Coatomer subunit beta-1
16	Solyc12g089240	Zinc finger protein CONSTANS-LIKE 3

(b)

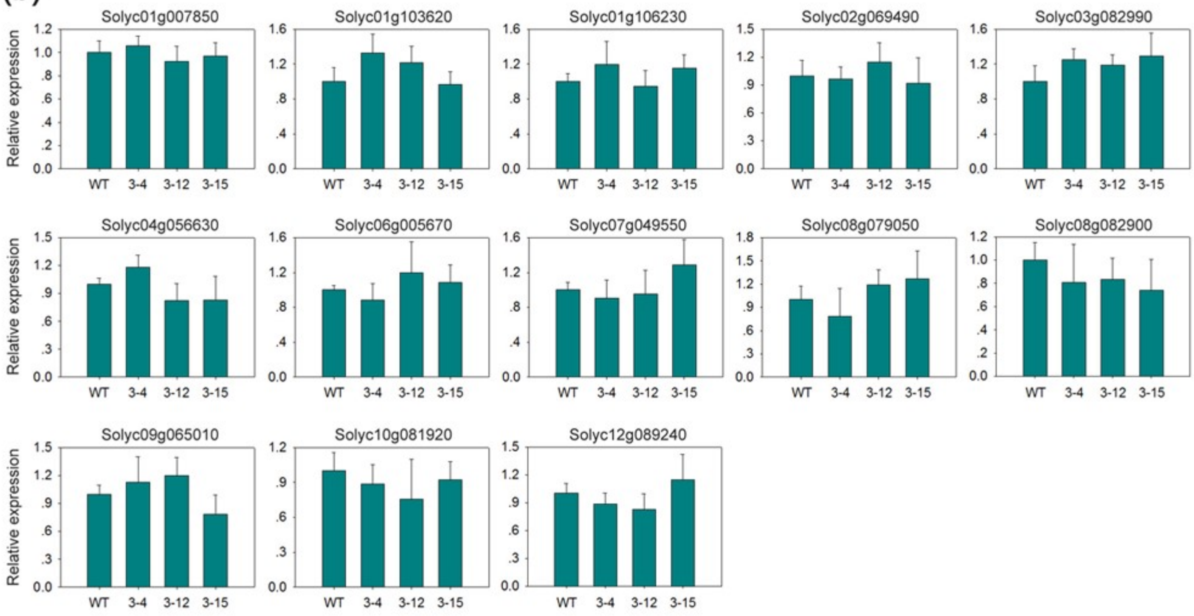

Additional file 4: Figure S2. Prediction of the potential off-targets of the RNAi construct. (a) The potential off-targets were predicted using the web-based computational tools pssRNAit [37]. The gene identifiers (Solyc numbers) and functional annotations are shown. (b) Expression analysis of the potential off-targets in leaves of wild-type (WT) and *SlVPE3* RNAi lines (3-4, 3-12, and 3-15) as determined by quantitative RT-PCR. The gene transcript levels are normalized against the *ACTIN* gene, followed by normalization against the WT expression. Values are means ± SD of three independent experiments. Transcripts corresponding to three genes (Solyc01g099000, Solyc03g071560, and Solyc03g077890) were not detected.

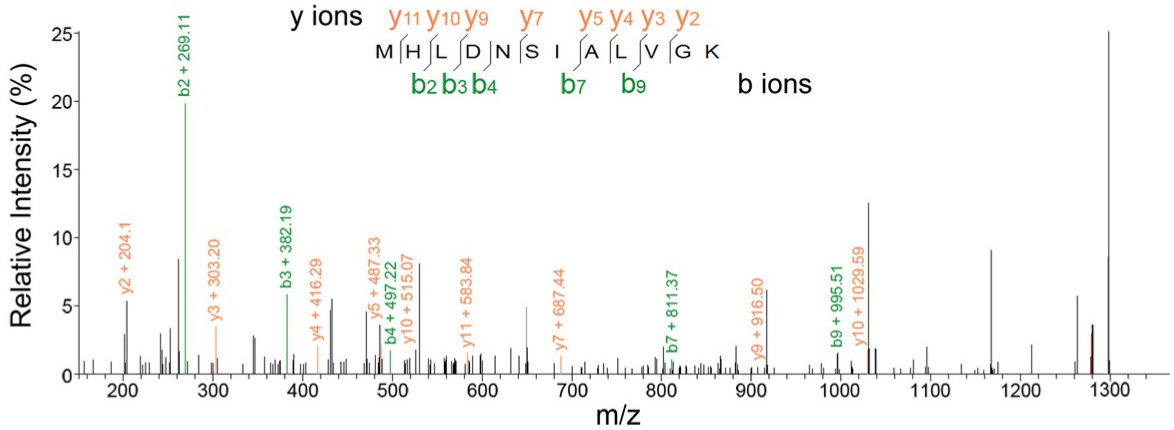

Additional file 4: Figure S3. Identification of SlVPE3 by mass spectrometry. Representative tandem mass spectra of peptide(s) identified from SlVPE3 are displayed. Sequences of identified peptides are shown above the mass spectra with b-ions and y-ions indicated.

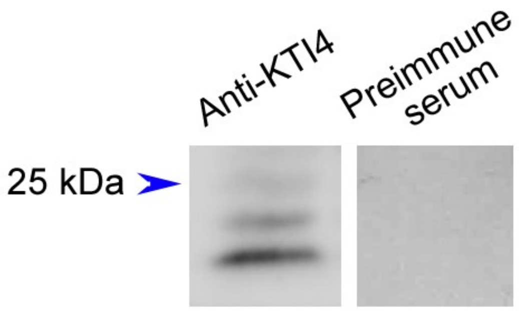

Additional file 4: Figure S4. Determination of KTI4 antibody specificity. Immunoblot analysis was performed using affinity-purified KTI4 antibody and perimmune serum. The predicted molecular mass of the full-length KTI4 protein appeared to be 25-kDa, indicated by a blue arrowhead.

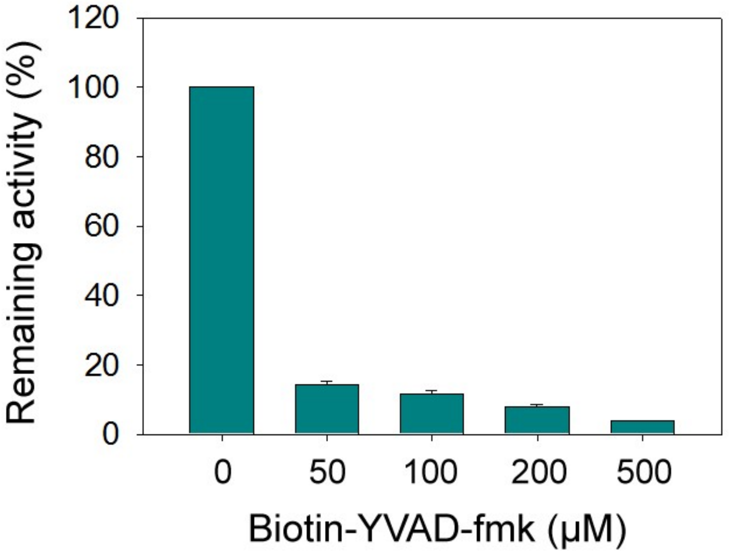

Additional file 4: Figure S5. Effects of biotin-YVAD-fmk on vacuolar processing enzyme (VPE) activity in tobacco (*N. benthamiana*) leaves transiently expressing *SlVPE3*. VPE activity was measured using a synthesized fluorescent substrate. Values are means ± SD of three independent experiments.

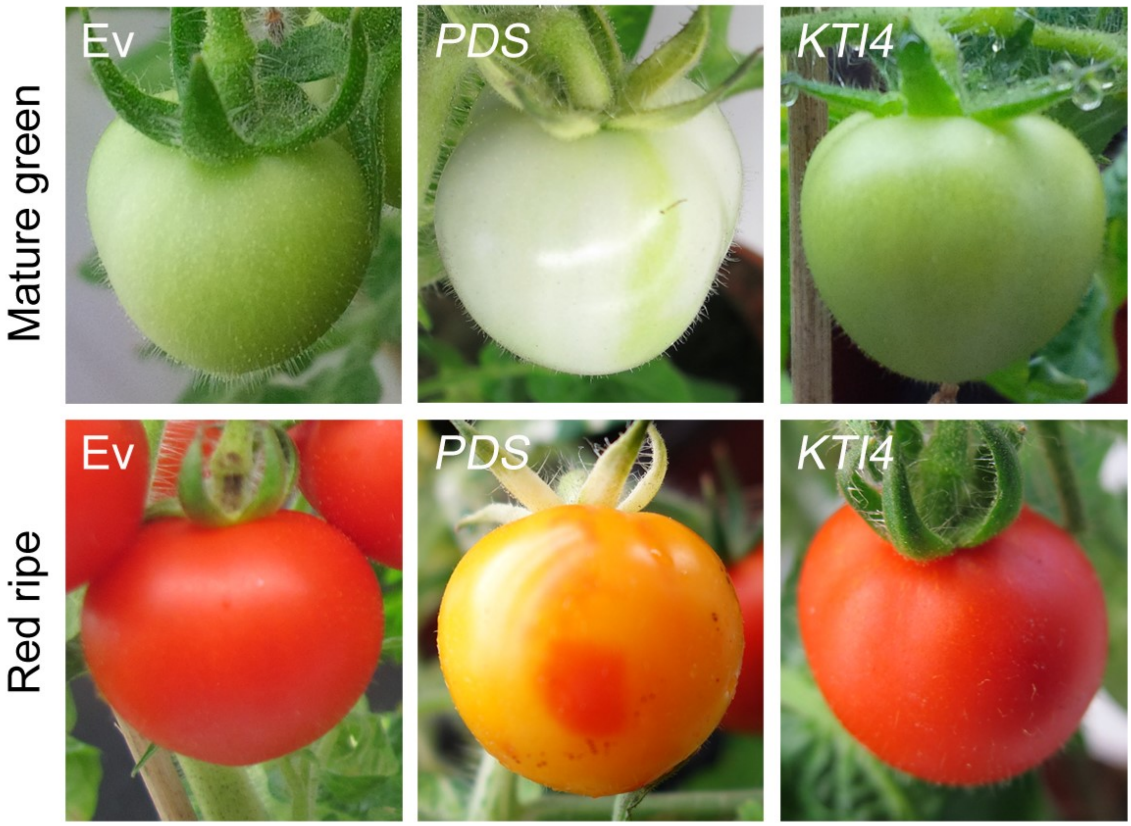

Additional file 4: Figure S6. Virus-induced gene silencing (VIGS) of *KTI4* in tomato has no effect on fruit ripening. Images show the fruit of plants infected with vectors containing no insert (Ev), specific *PHYTOENE DESATURASE* sequence (*PDS*), or specific *KTI4* sequence. Mature green stage fruit (upper panel) and red ripe stage fruit (lower panel) are shown.

Additional file 4: Supplementary text. Protein sequences of tomato vacuolar processing enzymes (VPEs).

>SlVPE1
MFVKINVASFLIALFVVLTEGRNVIERFDEDYEDSIGTKWAVLVAGSKEWYNYRHQANLCHAYQLL
KKGGLKDEHIIVFMYDDIANNPENPRPGVIINNPHGHDVYKGVPKDYTGKDCNAQNFYSVILGNK
SALTGGSGKVVNSGPNDYIFIYYTDHGAPGLVGMPEDPPVYAIDLNEVLKKKHASRTYKKMVFYL
EACDSGSMFADLLDEGLNIYATTSSKPDEDGWATYCYFTGDTSCYGECPPKDFKDNCLGDLFSV
SWLENSDLHDLQVETLEKQYLRIHKRVLNNGTHGSHMMQYGDLHINKDALSIYMGSNSPKHTSS
ANNNNASNSRHVNQRDVQLLYLISKFQNAPEGSRRKNEAYRKLSEVISEREHVDKSVKHIGQILF
GVENGQKVLNIVRQPLVDDWHCLKSFVKIFESHCGSLTSYGKKHIRGFANMCNAGIQRDQMDAA
AKQTCSS

>SlVPE2
MFAKINVASFLIILFVVLVEGSNMMESIFEDHENSIGTKWAVLVAGSNEWYNYRHQADICHAYQLL
KKGGLKDENIVVFMYDDIAYNSENPKPGVIINKPHGPDVYKGVPKDYTGKHCNAQNFYGVVLGN
KSALTGGSGKVVNSGPNDYIFIYYADHGGSGVIDMPIEPSIYAKDLNEVLKKKHASRTYEKMVFYL
EACESGSMFEGLPYKGLNIYVTTASKADENSYATYCSPKGYESTCLGDLFSVSWLENSELQDRQ
VETLKKQYQRIRKRVLNNGTEGSHMMEYGDLHIHEDALSIYMGSNFPTHTSSTKNNYASNSRHV
NQRDVQLLYLISKFQNAPEGSRRKSEAYRKLSEVILKREHVDNSVKHIGQILFGVENGPKVLNIVR
PAGQPLVDDWDCLKSFVKIFESHCGSLTRYGKKYVRGFANMCNAGIQRDQMDAAAKQTCSS

>SlVPE3
MNRSVAGVLFLIALSLNVSVSESRNFLKLPSEGSRFFDADEIDSVGTRWAILLAGSNGYWNYRH
QADICHAYQLLKKGGLKDENIVVFMYDDIANNEENPRQGVIINSPHGEDVYNGVPKDYTGDDVT
VDNFLAALLGNKTALTGGSGKVVDSGPNDHIFIFYSDHGGAGVLGMPTNPYLYANDLIDALKMKH
ASGTYKSLVFYLEACESGSMFEGLLPEGLNIYATTASNADESSWGTYCPGEYPSPPIEYDTCLGD
LYSISWMEDSERHNLRTESLKQQYHLVKERTASGNPAYGSHVMQYGDVHLSKDAVFLYMGTDP
ANDNSTFMDDNSLRVSKAVNQRDADLVHFWYKFHKAPEGSVSKTEAQKRLNEAISHRMHLDNS
IALVGKLLFGIKKGPEVLTSVRPAGQPLVDNWDCLKSYVRTFETHCGSLSQYGMKHMRSVANICN
AGIKMEQMVEASAQACPSVPSYTWSSLHRGFSA

>SlVPE4
MGSCNFTVCVTLMLLVMVGAISIEPKIDSRRLGRPHRFWDPLIRSPVDRDDDDETEEGGGGVR
WAVLVAGSNGYGNYRHQADVCHAYQILKRGGLNDENIVVFMYDDIAKSELNPRPGVIINHPNGS
DVYAGVPKDYTGEHVTAANLYAVLLGDKSAVKGGSGKVVNSGPNDRIFLYYSDHGGPGVLGMP
NMPYLYGKDLIEVLKKKYAARTYKEMVLYIEACESGSVFEGLMPENLNIYVMTASNAEESSWGTY
CPGMDPPPPSEYITCLGDLYSVAWMEDSESHNLKKETIKQQYEKVKERTSNSNNYNAGSHVME
YGSKEIKPEKVYLYQGFDPATVNLPANKIDFARLEVVNQRDADLLFLWERYKKLEDNSLEKAKLR
KEITETMLHRQHLDGSIDAVGVFLFGPIKGGSVLSSVRKPGLPLVDDWECLKSTVRLFEAHCGSL
TQYGMKHMRAFANICNNGISSDAMEDAFMAACNGHSLEEYTTANRGFSA

>SlVPE5
MVHVAGVFILVGIAVLAAVEGRNVLKLPSEASRFFDDADDSVGTRWAVLLAGSNGYWNYRHQAD

VCHAYQLLRKGGLKDENIIVFMYDDIAHHEENPRPGVIINSPAGEDVYEGVPKDYTGDDVNVHNF
LAVLLGNKTALTGGSGKVVNSGPNDHIFIFYSDHGGPGVLGMPTNPYLYADDLIAVLKKKHAAGT
YKSLVLYIEACESGSIFEGLLPNGLNIYATTASNAEESSWGTYCPGEYPSPPPEYETCLGDLYAVS
WMEDSEMHNLRTENLRQQYHLVKKRTANGNTAYGSHVMQFGDLQLSMESLFRFMGTNPANDN
YTYVDDNSLLASSKAVNQRDADLLHFWDKFRKAPEGSARKVEAQKQFTEAMSHRMHLDERIAL
VGKLLFGIQKGPEVLKHVRSAGQPLVDDWACLKSFVRTFESHCGSLSQYGMKHMRSIANICNAG
IQMEQMVEASAQACPSIPSNIWSSLHRGFSA

>SIVPE6
MIFKYKVCVALVVVLSICVNIEGRSVSKFLTEETVGTKWAVLVAGSNGWWNYRHQADVCLAYQLL
KKGGLKDENIFVFMYDDIANNTMNPRPGVIINNPHGQDVYKGVPKDYVGEDVNAENFFNVILAN
KSGITGGSGKVLNSDPNDHIFIYYVDHGGPGIVSMPTGVVYANDLIDVLKKKLGSGTYSKLVFYLE
ACEYGSMFDGLLPEGLDIYVTTASNPNESSWGTYCGVGDARDPCLVACPPPEFKVMFMIDKLKL
WMIIMFHYDQIADRTAANLTYGSHVMQYGDMLLSVDALFQYMGVASINHSHVSMNSYKSSSQN
VERRETELFYWQSKYDDAPEGSDDYFEARAKLINVVAHRSQVDNNVKHIGDLLFGVKYGNEAL
QTVRSSGQPLVDNWDCLKSYVEIFEAHCGKLSSYGKKHIRGIANICNAGIEREQMTAATVQACGP
L

>SIVPE7
MISRYNIVGIFFLVLVSIVVTIEGRSITQFLTQKTKGTKWVVLVAGSNGWTNYRHQANVCHAYQIM
KAGGLKDENIIVFMYDDIANNTENPRPGVIINNPHGHDVYKGVPKDYVGDDVNANNFFNVILANK
SGVVGGSGKVLKSGPNDHIFIYFTDHGAPGVISMPYNEVIYAHELVNMLKKKHASGTYDRLVFYL
EACESGSMFDGILPKGLNIYAMTASKPDEDSYGTYCGESTPVDSCWGQCPPPEFKGVCLGDLF
NVSWMEDSDVQDRKTNSLHQQYSRVAKRTAANLTYHNYGSHVQEYGDKVMSFDPLAAYLGET
SKNHSHNSVDAKSFSTSSSRNVDQRSTELFYLFTKHQNAPEGSDEKYEAKVKLNELMSQRSQV
DSNVKHLGDLLFGVEKGNEVLHSVRPAGKPLVDNWDCLKSYVKIFETHCGRLTVYGRKHVRGIA
NICNAGITSEKMAAMSAQACSS

>SIVPE8
MISRYNIGVFFLVLVSISVNTEGHSISQFLTRKTKGTKWAVLVAGSRGWINYRHQADVCHAYQILK
VGGLKDENIIVFMYDDIANNTANPRPGVIINNPHGHNVYKGVPKDYVGDDVNAKNFFNVILANKS
GVVGGSGKVLKSGPNDHIFIYYTDHGSPGMIIMPSGEPIYADELFNVLKKKHASKTYDKLVFYLEA
CESGSIFDGILPKGLNIYAMTASKPNEDSFGTYCGESTPIDSCWGQCPPPEFKGLCLGDLFSVAW
MEDSDVQDRKTNTLHGQYIRVAKRTAANLTYDKYGSHVKEYGDKVVSFDPLVVYMGETSKNHS
HDSVDAKSFSTSSSRNVDQRSTELFYLVIKHQNAPEGSDEKYEARVKLNEVMSQRSQVDNNVK
HLADLLFGVEKGNEILHSVRPAGQPLVDNWDCLKSYVKIFETHCGRLTVYGRKHVRGIANICNAG
ITSEKMDAMSAQACSS

>SIVPE9
MISRYNIVGVFFLVLISIFVNIEGRSISKFLTQKTKVTEWAVIIVGSAGWTNYRHQANVCHTYQIPKI
DGLKDENIIVFIDLVSSSITHMAMMFTKDYVGDDVNAKNLFSVILANRSGVVGRSGKVLKSGQMTI
SSYTMLIMVPLDLFFFNFDIPYAILTPQDQKVLNFVTLKLKLAICDTLKLIRICITIFLFLLLSTMPSGE
LIYADELFDVLKKKHASGTYDRLVIDLEVCESGSMFDGIPPKGLNIYAMTVAKRTAANLTYRNYGS
HVHKYGDKVVVFDPLAAYMGETSKNHSHNSLDAKPFSTSSSRNVDQCSTELFYLFTKKSQVDS

NVKHLGELLLGVEKGNEVLHTVRSAEQPLVDNWDCLKSYVKIFEAHCGRLTAYGRKHVRGIASIC
NAGITSEKMAAMSAQAFST

>SIVPE10
MIFKYIVCVALVVVLSICVNIEGRSVSKFLTEETVGTKWAVLVAGSNGWWNYRHQADVCHAYQLL
KKGGLKDENIIVFMYDDIANNTMNPRPGVIINNPHGQDVYKGVPKDYVGEDVNAENFFNVILANK
SGITGGSGKVLNSAPNDHIFIYYVDHGGPGIVSMPTGVVYANDLIDVLKKKHGSGTYSKLVFYLEA
CESGSMFDGLLPEGLDIYVTTASNPNESSWGTYCGVGDARDPCLVACPPPEFKGVCLGDLYSV
AWMEDSDVQDRQTETLDDQYDRIANRTAANLTYGSHVMQYGDMVLSVDALFQYMGVASINHS
HVSMNSYKSSSQNVEQRETELFYWQSKYDNAPEGSDDYFEARAKLINVVAHRSQVDNNVKHIG
DLLFGVKYGNEALQTVRSSGQPLVDNWDCLKSYVEIFEAHCGKLSSYGKKHIRGIANICNAGIER
EQMTAATVQACGPL

>SIVPE11
MISWYNIIVIFLFGLVSIFVNIEGRSISQFLNQESQGTKWAVLVAGSNGWDNYRHQADVCHAYQLL
KNGGLKDENIIVFMYDDIAHNRENPRPGVIINNPHGNDVYKGVPKDYVGEDVNALNFYNVILANK
SGIVGGTGKVLNSGPNDHIFIYYTDHGGPGIVAMPSGELVYANDLVNVLKKKHASGTYDRLVFYL
EACESGSMFDGLLPEGLDIYVMTASEPNEDSWATYCGEGTPEEPCLVQCSPPEFQGVCLGDLY
SISWMEDSDIQDRTADSVQGQYSRWKIRFMFFILRPKVANRTAANITHGSYGSHVTEYGDIVVSF
DSLAAYMGENFKNHSHDSVDAKSFSTSSSRNVDQHSTELFYLFAKHRKAPEGSNEKYEALVKLN
EVKSQRSQVDYNVKHLGELLFDVEKGNEVLNSVRPARQPLVDNWDCLKSYVKIFEAHCGRLTTY
GRRHVRGIANICNAGITNEKMVAMSAQACSS

>SIVPE12
MIYRDYIVGVFFLVLVSIFVNIESRSISQFLTQKTKGTKWAVLIAGSAGWTNYRHQVDVCHAYQVL
KAGGLKEENIIVFMYDDIANNTKNPRPGVIINNRQGHDVYKGVPKDYVGDDVNAKNLFNVILANK
SGVVGGSGKVLRSAMPSGELIYADELFNMLKKKHASGTYDRLVIYLEACESGSMFDGILPKGLNI
YAMTASKPDENSFATYCGDGTPDHPCFGPCSLPEFKGICLGDLYSVAWMEDRTAANLTYGSHV
MQYGDLMVSFDPLATYMGENFKNHSDDSVDAKSFSTSSSRNVDQRNSELFYLFTKHQKALEGS
DEKYEALVKLNKVKSQRSQVDNNVKHLGELLFGVEKGNEVLNNVRPAGKPLVDNWDCLKSYVKI
FEAHCGRLTTYGRRHVRGIANICNAEITNEKMVSMSSQACSS

>SIVPE13
MISRCNIVGVFFLVLVSIFVNIEGRSISQFLIQKTKGTKWAVLVARSNGWTNYRHQADVCHAYQILK
AGGLKDENIIVFMYDDIANNTENPKPGAIINNPHGHDVYKGVPKDYVGEDVNANNFYNVLLANKS
GVIGGSGKVLKSGPNDHIFIYYTDHGAAGFITMPSGESIYADDLFNVLKKKHASGMYDRLVFYLEA
CESGSMFDGIFHKGLNIYAITASKPDENSFGTYCGDGTPDDPCFGQCPPPEFKGVCLGDLFSVA
WMEDSDVQDRKINSLHGQYSRVAKRTAANLTHHNYGSHVQEYGDKVVSFDPLAAYMGETSKN
HSHDSVDAKSFSTLSSRNVDQRSAELFYMFTKRSQVDNNVKHLGELLFGVEKGNEVLHTVRRA
GQPLVDNWDCLKSYVKIFEAHCGRLTVYGRKHVRSIAKICNAGITSEKMDAMSAQACSS

>SIVPE14
MISRYNIVGVFFLVLVSIFVNIEGRSISQFLAQKTKGTKWAVLIAGSAGWTNYRHQADVCHAYQILK
TGGLKDENIIVFMYDDIANNIENPRPGVIINNPHGHDVYKGVPKDYVGEDVNAINLFNVILANKSG

VVGGSGKVLKSGPNDHIFIYYADHGAPGFISMPSGELIYAHELFNVLKKKHASGTYDRLVIYLEAC
ESGSMFDGILPKGLNIYAMTASKPDEASFGTYCGNGTSHTPCFGQCSPLEFKGICLGDLYSVAW
MEDRTAANLTYRNYGSHVQEYGDLVVSFDPLVAYMGETSKNHSHDSVDAKSFSTSSSRNVDKR
STELFYLFTKHENAPEGSDEKYDALVKLNEVMSHRSQVDNNVKHLGELLFGVEKGNEVLHTVRP
AGQPLVDNWDCLKSYVKIFEAHCGRLTAYGKKHVRGIANICNAGITSKKMAAMSARACSN
